# Eight Hypotheses on Technology Use and Psychosocial Wellbeing: A Bicultural Phenomenological Study of Gaming during the COVID-19 Pandemic

**DOI:** 10.1007/s12144-022-03586-x

**Published:** 2022-08-22

**Authors:** Veli-Matti Karhulahti, Henri Nerg, Tanja Laitinen, Antti Päivinen, Yingrong Chen

**Affiliations:** Faculty of Humanities and Social Sciences, Department of Music, Art and Culture Studies, University of Jyväskylä, PO Box 35, FI-40014 Jyväskylä, Finland

**Keywords:** Covid-19, Gaming, Qualitative methods, Technology use, Wellbeing

## Abstract

In this nonconfirmatory qualitative study, we pursued a range of hypotheses regarding how gaming operates in the lives and psychosocial wellbeing of those who actively play videogames during a crisis, such as the COVID-19 pandemic in 2020. Informed by an explorative survey (*N* = 793), interpretive phenomenological analysis was applied to interview data from actively gaming Chinese (*n* = 10) and Finnish (*n* = 10) participants. Our findings demonstrate how the general increase of pandemic-time gaming did not manifest in all player groups, but in some life contexts gaming activity rather decreased along with reformations in subjective meaning hierarchies and values. Ultimately, eight subordinate themes were refined into testable hypotheses. The study encourages policies that promote socially supportive gaming during pandemic-like situations to consider including personally meaningful solitary play in their recommendations and highlighting context-specificity over generalization. Finally, as almost all our data points echoing experiences of decreasing gaming activity came from China, we stress the importance of culturally diverse samples in the psychological study of global phenomena.

**Supplementary Information:**

The online version contains supplementary material available at 10.1007/s12144-022-03586-x.

## Introduction

As the COVID-19 pandemic spread globally in 2020, people around the world were forced to stay home more than ever before. At the same time, discussion about media use causing and preventing health problems intensified. Especially gaming was considered both a risk and a solution to the new challenges of wellbeing. Along with its warnings about too much playing time, the World Health Organization also encouraged people to play during the pandemic (Chambers, 2020; WHO, 2020). Only a year after the beginning of the pandemic, several survey studies had already been published regarding gaming and wellbeing, collectively indicating videogames to serve as neutral or supportive life components during social restrictions (Almomani et al., 2021; Balhara et al., 2020; Barr & Copeland-Stewart, 2021; De Pasquale et al., 2021; Gabbiadini et al., 2020; Giardina et al., 2021a; Johannes et al., 2021a). It remains unknown, however, how players of videogames *experienced* the pandemic and what psychosocial functions gaming carried in these lockdown experiences. To fill this research gap, the present qualitative study was set up to explore how actively gaming people experienced videogames as part of their daily lives during lockdown.

Because the COVID-19 pandemic was unique in terms of global social restrictions, to our knowledge, previous psychological research on the phenomenology of lockdown play does not exist. Nonetheless, studies regarding gaming experiences during pre-pandemic times have yielded findings, which expectedly resonate with lockdown play. For instance, several qualitative studies (e.g., Iacovides & Mekler, 2019; Shi et al., 2019; Snodgrass et al., 2014) have demonstrated how gaming is sometimes utilized as a coping mechanism for stress and other life difficulties. It is likely that the pandemic triggers similar coping responses in some players. Because the prolonged pandemic represents mostly unforeseen causal chains leading to potential stress, coping-by-gaming in socioculturally diverse lockdown situations remains an unmapped area of qualitative psychological interest, however.

Numerous in-depth studies have also documented the varying social functions of gaming across life contexts (e.g., Chen, 2012; Fine, 1983; for a review, see Bowman et al., 2022). That said, it remains unknown how those social functions were affected by the pandemic. Although multiplayer gaming with social features clearly increased in total quantity during times of social restrictions (Vuorre et al., 2021), it is not known if and how the *nature* of social interaction in videogames changed, or how the effect heterogeneity (of various wellbeing-related constructs) manifest in individual cases (Johannes et al., 2021b). Different changes in social dynamics can be expected to occur in people’s gaming routines during lockdown (see Domahidi & Quandt, 2015).

Finally, following the World Health Organization’s recent decision to recognize “gaming disorder” as a diagnosable mental disorder, which remains controversial among experts (Ferguson & Colwell, 2020), it is also relevant to address the official warnings regarding how excessive gaming during the pandemic “may lead to the development of gaming disorder” (WHO, 2020). We found one brief quantitative report (Higuchi et al., 2020) with participants who had sought treatment for gaming-related health problems, suggesting prolonged use of the internet and gaming arising out of the COVID-19 pandemic. On the other hand, when it comes to how such increased media use affected the participants’ wellbeing and life balance, the authors report mixed results with several measured health problem areas having, in fact, improved along with increased gaming. To better understand the complex dynamics between gaming and other daily behaviors in different experiential contexts, nonconfirmatory approaches are needed with idiographic focus on individual lives. With such phenomenological motivations, we ask the following:

- **RQ1**: How do actively gaming individuals experience the COVID-19 pandemic and videogame play during it?

Our leading research question is further motivated by the overarching meta-goal of cumulative science. Currently, the vast heterogeneity of gaming behaviors severely complicates related correlational findings, which are often carried out deductively by instruments that measure constructs with predefined relevance. In other words, hypothesis testing in psychology is often theory driven, but as the ongoing theory crisis has made manifest (e.g., Eronen & Bringmann, 2021; Fried, 2020; Maatman, 2021), theories that are genuinely falsifiable, operationalizable, and useful do not exist in excess. Hence, it can be useful to pursue hypotheses development with explorative qualitative methods, which avoid many of the confirmatory biases attached to dominant paradigms (e.g., Feyerabend, 1993), especially in theoretically undeveloped psychological subfields such as that of gaming (Karhulahti, 2022). Accordingly, our study was designed so that its phenomenological findings could be refined into concrete, testable hypotheses.

To combat the strong western bias in previous literature, we decided to carry out our study as a bicultural enterprise with Chinese and Finnish participants. To assist, inform, and contextualize our upcoming qualitative work, we also carried out a survey in the two countries (Appendix 1), which produced a second research question.

- **RQ2**: How do people decrease, maintain, or increase their gaming during the pandemic?

In sum, our goal was to exploratively pursue new phenomenologically sourced hypotheses regarding how gaming operates in the lives and psychosocial wellbeing of those who actively play videogames during a crisis, such as the COVID-19 pandemic, with a secondary interest in how decreasing, maintaining, and increasing gaming habits are associated with these lived experiences.

## Materials and Methods

**Fig. 1:**
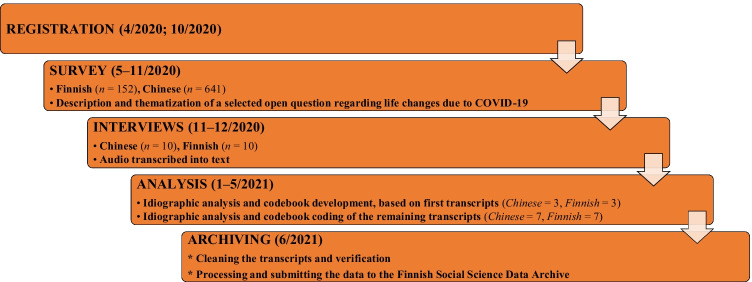
A workflow diagram of the study

Research planning started in spring 2020, as COVID-19 had just been declared a pandemic. Finland and China were selected as countries for data generation because they provided much-needed cultural variety and the research team had native linguistic expertise in both. To assist, inform, and contextualize our qualitative findings—*which are not general but specific*—we additionally distributed a survey regarding the potential changes in the players’ behaviors and everyday activities in the studied countries. These secondary survey data are not analyzed here but described (Appendix 1), following the best practice of reporting all collected data and analyses (Simmons et al., 2011). The data generation plans were registered on April 2, 2020, locally, allowing the study to receive a small amount of financial support, and a preregistration was uploaded to the Open Science Framework on October 31, 2020 (see Supplementary material). The study followed the guidelines of the Finnish National Board on Research Integrity, according to which a separate ethics review should not be applied. The workflow is presented below (Fig. 1).

### Method

Our methodological basis for the interviews and their investigation is interpretative phenomenological analysis (IPA; Smith et al., 2009). For almost three decades, the IPA method has been found to be useful in psychology for investigating how people understand their own felt and lived experiences, and how are they meaningful (Smith et al., 2009). This phenomenological investigation is double hermeneutic in the sense that researchers *interpret* what the interviewees have *interpreted* of themselves. Additionally, a codebook was developed and applied to support the analyses.

### Participants

Participants had to be a minimum of 18 years of age and have gaming as part of their weekly activity. Participants were excluded if they had severe disorders or health conditions. This criterion was set because the IPA pursues specificity over generality (e.g., Larkin & Thompson, 2012). In IPA, better results are reached with homogenous samples, and including clinically complex health scenarios would not have improved the design. The general IPA sample size recommendations differ between *N* = 1 and *N* = 15, depending on the topic and context of analysis (see Smith & Osborne, 2007). Unlike those qualitative approaches that justify their samples by saturation, IPA typically operates with the limits set by the topic and its context. Because our goal was to carry out a bicultural study with both Chinese and Finnish participants, we pursued a higher sample size than recommended. At the time of the interviews, both countries still had major restrictions for meeting people and moving in public spaces due to the pandemic. The survey process allowed us to recruit participants via random selection: we contacted those who had expressed interest in interviews, with the only consideration being gender balance. We did not screen changes in playing habits, but the ratio between those who had decreased, maintained, and increased gaming happened to be in line with the survey results (Table 1; Appendix 1). Our goal was not to compare the two countries, but to incorporate experiences from diverse cultural contexts.

**Table 1:** Selected (abstracted) participant details (*N* = 20). Gaming activity * = decreased, ** = maintained, *** = increased

	Age	Occupation	Household	Country	Gender
1***	20–25	From customer manager to broadcast media	Lives with parents	China	Man
2**	20–25	Manager assistant	Lives alone	China	Woman
3*	20–25	Security guard, game companion	Lives with parents	China	Man
4***	20–25	Graphic designer, game companion	Lives alone	China	Woman
5**	20–25	Waitress	Lives with partner	China	Woman
6**	20–25	Telemarketer	Lives with/near partner	China	Man
7***	< 20	Digital artist, student	Lives with roommates	China	Woman
8**	25–30	Sales, part-time online sales	Lives alone	China	Woman
9***	20–25	Previously clerk, now unemployed	Lives alone	China	Man
10*	20–25	Unemployed, part-time sales	Lives alone	China	Man
11***	25–30	Student, part-time teacher	Lives with partner	Finland	Woman
12***	30–35	From healthcare work to student	Lives with family/children	Finland	Woman
13***	20–25	Fulltime student	Lives alone	Finland	Man
14***	30–35	Healthcare work	Lives with family/children	Finland	Woman
15**	25–30	Remote work (unspecified)	Lives alone	Finland	Woman
16***	20–25	Unemployed, starting to work	Lives alone	Finland	Woman
17**	20–25	Fulltime student	Lives alone	Finland	Woman
18***	25–30	Office worker	Lives alone	Finland	Woman
19***	30–35	Teacher	Lives alone	Finland	Woman
20**	20–25	Student, starting to work	Lives alone	Finland	Man

### Procedures

The interviews took place between November and December in 2020, and interview length varied between 60 and 90 min (for an outline of topics, see Supplement 1). The Finnish interviews were conducted remotely with two team members using Zoom software (first three interviews VMK & HN, other interviews HN & AP). The interviews were transcribed manually into text word-by-word by our team members (HN, TL, AP), after which the audio was deleted. The transcripts were further proofread and anonymized (VMK), which enabled us to store them for open scientific use in the Finnish Social Science Data Archive (FSD). The Chinese interviews were carried out by our Chinese team member (YC), following similar procedures. Due to our team only having one Chinese member, we did not have the resources to (back) translate these interviews verbatim. Instead, the interviewing researcher produced three- to four-page English transcripts with core contents for the team to enable internal discussion but using the original Chinese data for coding and interpretation. We did not seek consent from the Chinese participants to process their interviews for reuse (FSD can process only English, Finnish, and Swedish data).

### Analysis

The analysis followed an applied IPA approach: we combined phenomenological interpretation with team-based (five members) open thematic cross-case coding. In other words, both IPA and open coding were carried out in parallel. The analytical process proceeded in the following order.

1. The team gathered for two training sessions where coding procedures were synchronized. Two researchers were selected for IPA (interviewing and non-interviewing) and open coding (interviewing and non-interviewing) with each transcript, that is, there were four coders in total (HN, TL, AP, YC).
2. Initially, three Finnish and three Chinese transcripts were interpreted and coded openly. In the case of the Chinese interviews, this phase was limited by non-Chinese researchers using the English summaries. The initial IPA themes were shared with 200-word verbal descriptions and the open codes were shared as lists with the help of ATLAS.ti.
3. All of the team members read and reread the six (3 + 3) transcripts and shared interpretations, and two meetings were held for the team to discuss and negotiate subordinate IPA themes as well as general thematic patterns, which were turned into a codebook (Supplement 2).
4. The codebook was then used by those conducting non-IPA coding to find detailed instances of *emotional*, *social*, and *life during covid* codes (selective coding) in the rest of the transcripts. Idiographic IPA was carried out, but the codebook assisted in comparing subordinate themes concerning the *emotional*, *social*, and *life during covid* data domains.
5. After dual analysis of the remaining 14 (7 + 7) transcripts, the lived experiences of the participants were discussed over three new meetings to negotiate discrepancies until agreement. Following RQ2, overlapping superordinate IPA themes were organized according to decreased, maintained, and increased gaming. Relevant open codes were turned into tables.

**Fig. 2:**
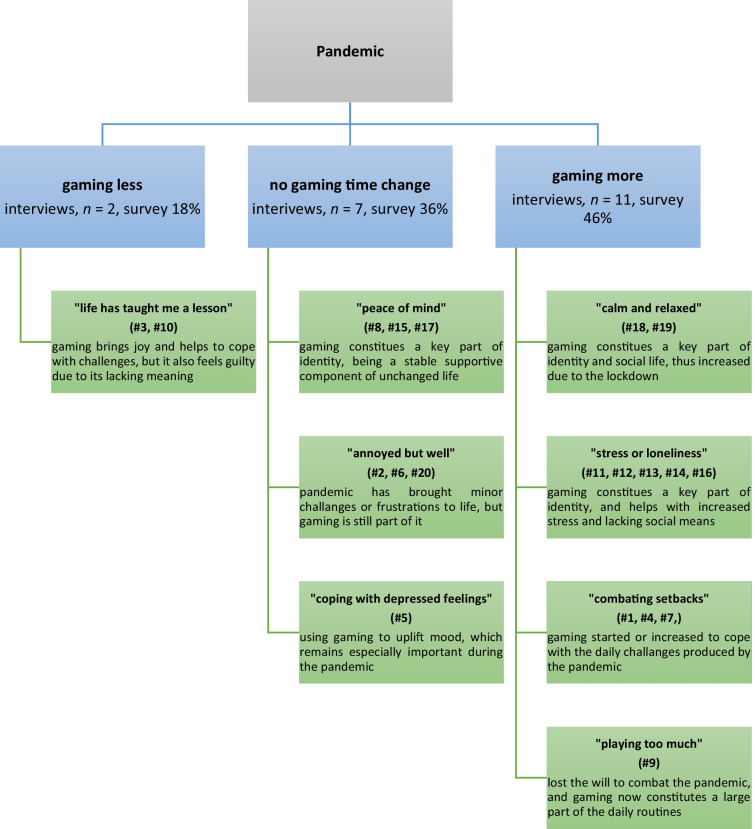
Eight subordinate themes of COVID-19 experiences based on the interviews (*N* = 20)

A summary of the participants’ emotions, experiences and meanings is provided in Table 2. Subordinate themes are presented in Fig. 2. With respect to the flexibility of IPA and our goal to refine the findings into testable hypotheses, we aimed at synthesizing the results into a form that is more applicable and pragmatic. As an unexpected side product of our analysis, we discovered a great variety of how the participants experienced “social gaming,” which we report separately (Appendix 2).

**Table 2:** A summary of the participants’ (*N* = 20) experiences during the pandemic. 7DtT = *Seven Days to Die*, CSGO = *Counter Strike: Global Offensive*, HoK = *Honor of Kings* (王者荣耀), LoL = *League of Legends*

	Emotional State	Changes in Life	Videogames Played	Gaming Experiences… Function/Meaning	
1	Challenged and struggling	Unemployment followed by a new broadcast job	HoK, PUBG	Competitive excitement; social joy	A regular means for escapism
2	Uncertain and yearning	Salary suspended; increase in social interaction	HoK	Feeling skilled and meeting new people	A core particle of daily life routines
3	Guilty and lonely but hopeful	Grandparents ill and parents unemployed; new gaming-related job	HoK, PUBG, QQ Speed	Previously feelings of mastery and skill, now social exploration	Polar: occupation, private (socially significant)
4	Bored and uncertain	Work suspended, new gaming-related job; more time for friends/family	HoK, QQ Speed	Agency in a private space (when not playing to work)	Polar: occupation, private (calming routines)
5	Depressed and lonely	Salary suspended; valuable social meetings cancelled	HoK, PUBG	Social satisfaction	Tool to cope with lacking social life
6	Frustrated but relaxed	Some social events cancelled	HoK	Social exploration	Deepening of social bonds
7	Blue and downhearted	A good friend passed away	HoK	Relaxing alone, but harassed by toxicity	Personal place of comfort
8	Happy and fortunate	Salary was cut down, but a new online sales job	Love & Producer, Love Nikki, Dragon Raja	Romantic fantasy	Identity particle; parasocial bonds
9	Lack of motivation and energy, feeling numb	The company he worked for was shut down	Crossfire, HoK	Solitary pleasures of absorption, progress	A hiding place from life issues
10	Anxious, “awaken” to a reality	Unemployment followed by a new job	HoK	Personal achievement and social exploration	Tool to cope with negative emotions
11	Stressed and worried, but well	Diminished social routines; controlling news intake	Borderlands 3, Harry Potter: Wizards Unite	Thrill of achievement and completion	Identity particle; routine-supportive
12	Lonely and strained, but generally satisfied	Diminished social routines, but children are more at home; studies remotely	Pokémon Masters, Hay Day, Zombies Run!	Committed to achieve daily goals; absorbed in stories/development	Identity particle; tool for relaxation and socializing
13	Lack of motivation, isolated, and uncertain	Studying became remote, physical hobbies and social events all cancelled	Among Us, Minecraft, CSGO, Hearts of Iron 4	Explorative freedom and planning; hanging out with friends	Identity particle; coping tool for lacking social life
14	Stressed and uncertain	Important travels and social events cancelled	I Love Hue, Minecraft, Growtopia, Monument Valley, Push Sushi	Aesthetic beauty, order, and control; bonding with family	Identity particle; coping tool for mood and stress
15	Peace of mind and happiness	A new remote job; more free time and energy; many new hobbies and projects	Beat Saber, Skyrim, Fallout 4, Spyro the Reignited Trilogy	Sensing audiovisual beauty; accomplishing and learning things	Basis of identity; bad days remedy and exercise tool
16	Frustrated, but mood swings quickly	A new job; time spent with friends turned completely virtual; knits when stressed	Among Us, LoL, World of Warcraft, Tabletop Simulator	Sharing good moments with others; having diverse “fun”	Identity particle; a means for being with friends
17	Occasional boredom, but overall content	Spontaneous social life is no more possible; board gaming has turned virtual	Monster Hunter, Noita, Mount & Blade 2, Tabletop Simulator	Feelings of achievement and cooperation	Identity particle; provides sense of progression
18	Calm and relaxed	Work now remote; more time for self and friends	CSGO, Walking Dead: Our World, Fruit Ninja	Sharing moments with others; routine joy	Identity particle; basis for routines
19	Happy; empathizes for those who are less	Musical hobbies cancelled but more work; less opportunities with friends	Animal Crossing, Final Fantasy 7, Dungeons & Dragons	Story immersion; positive escape via learning/success	Identity particle; brings a sense of content/structure
20	Frustrated but well	Studies turned remote and moved to a new job; key social events cancelled	Minecraft, War Thunder, Rising Storm 2, CSGO, 7DtD	Feelings of excitement and cooperation	A core particle of daily life routines and social life

## Results

All participants were analyzed with equal depth (narrative summaries in Supplement 3), which produced eight subordinate themes: “life has taught me a lesson,” “peace of mind,” “annoyed but well,” “coping with depressed feelings,” “calm and relaxed,” “stress or loneliness,” “combating setbacks,” and “playing too much.” All subordinate themes and their derivative hypotheses are addressed in the discussion below. Following the idiographic standards of IPA reporting, one participant/transcript was selected randomly from each type of habit change (maintained, decreased, or increased gaming activity) for more detailed presentation. The participants are referred to by invented pseudonyms and their quotes are translated to English.

### Maintained gaming activity: Anna (#15), “peace of mind”

Anna is a woman who lives alone. After studying and working part-time for many years, she started her first full time job at the beginning of the pandemic. Anna works remotely, which fits her well. She self-identifies as an introvert, and gaming has always been a central part of her life. Her late father was a gamer, and she used to play with him regularly. In her upper secondary school years, Anna started playing *Guild Wars* (an online roleplaying game) with her friend and they played it “six years straight,” after which she has avoided playing similar videogames due to being easily “hooked.” Perhaps because of the above, she now keeps her gaming generally to four hours per day and mostly plays single-player games, as her friends have families and other interests.

Anna feels that the pandemic has improved her life quality in general. Before the pandemic, she was pressured to attend social events she does not enjoy, which would lead her to come up with excuses to avoid them. Now, as all social events are cancelled, Anna’s previous cognitive dissonance has resolved: she is free from other people’s expectations and has “peace of mind,” being able to focus on the things that truly matter to her. Gaming is one of these things; however, as she was already playing several daily hours before the pandemic (“I’ve always played a lot”), she does not play more (“this was a normal year”). She rather uses the extra hours on things that she did not have time for before, such as cooking and handicrafts.

Gaming is part of Anna’s daily routines outside working hours. She goes to work in the morning and plays many different videogames in the evening or nighttime. She also has a virtual reality setup, which she uses to exercise by playing *Beat Saber*. Anna likes open world roleplaying games, such as *Skyrim* and, currently, *Borderlands 2*—she usually has three or four videogames “in the works.” For her, aesthetic beauty is a key part of the gaming experience.

*Anna*: The graphical appearance is very important. That’s how I first get interested in a videogame… it must have something that looks memorable, arouses feelings, or thoughts. For instance, when I played *Skyrim*, I often stopped for long periods of time to just admire the landscape, environment, everything was beautiful.

*Interviewer*: So, you literally just stop for the view?

*Anna*: Yes, quite often. And sometimes I know there will be a nice view somewhere, so then I look for those places. In *Skyrim*, I felt a need to climb the tall mountains because I knew it would look beautiful there.

This “beauty,” including audio and music elements, is the essence of the virtual environment in which Anna feels comfortable and happy—a horror or dark-themed videogame would not work. However, she also expects the videogames to reward and satisfy her. Especially when she feels down or the day at work has not been a good one, she wants to feel triumph in a beautiful videogame. Videogames provide Anna a place of comfort where she is safe and satisfied.

*Anna*: The videogame must challenge me positively, not so that “I cannot take this,” it can be too much. I seek good experiences, experiences of success. I like when I succeed.

*Interviewer*: How would you further describe this “success”?

*Anna*: Well, it’s like when I get over with a big boss fight or complete a difficult quest. There are many things involved… But it’s just that when I’ve had a bad day at work, I need to accomplish something, and then I take one these videogames, and it rewards me.

Gaming is clearly a central part of Anna’s history and identity. She grew up with her friends and parents playing videogames, and she still talks regularly about her gaming to her mother who “digs” it: “I had to text her that this videogame [a remake of *Spyro* that she played as a child] is just so cool and nostalgic.” As Anna’s friends “grew out of videogames,” she kept gaming, which still defines who she is. Gaming, moreover, enables Anna to maintain a positive mood and expand her curiosity for novel projects.

*Anna*: The most important thing for me is to keep learning new things [in gaming and elsewhere], I must have many projects ongoing and not all of them ever get finished. But I always learn something new and that makes me better prepared for the future.

As domestic gaming represents Anna’s past, present, and the future—with many routinized emotional and motivational dimensions—the pandemic had hardly any influence on her playing routines.

### Decreased gaming activity: Jingfan (#10), “life has taught me a lesson”

Jingfan lives alone in a city that had a high infection rate during the pandemic, which led to the shutdown of the local company that he worked for. He was left unemployed, which in turn led to financial difficulties and forced him to look for a job that could be done remotely. In general, the pandemic made him feel suffocated, as social interaction and movement were restricted. Among other things, the usual visits to his parents had to be cancelled.

*Jingfan*: The whole city was under quarantine, and even though I have never been actively going out to socialize, there is a difference between “I want to go out but I cannot” and “I don’t want to go out.” It [*I want to go out but I cannot*] is like being held in prison. I felt that I had lost my freedom.

Before the pandemic, Jingfan often played until late at night, mainly single-player games. Playing with others that he knew made him feel awkward, as this forced him to be “considerate about their gaming experience,” which again limited his own experience. With those close to him, he could not play in the way he wanted, but in a way that would satisfy them and assure that their experience was enjoyable. In the same way as Jingfan felt the pandemic was keeping him “imprisoned” even though he did not go out much, having his family or friends play with him “imprisoned” his gaming experience even though he could not say how being “considerate” manifested in practice. The *feelings* of independence and not being influenced by others are crucial to his gaming experience.

Having lost his job to the pandemic, Jingfan felt that he had learned to respect other things in life. Instead of spending time and money on entertainment, such as gaming, he now had new priorities grounded in practical matters.

*Jingfan*: Today I feel that I should have saved some money. My situation would be much better now. When something like this happens unexpectedly in your life, nobody can help you, you must be ready to take care of yourself. I know so many people who lost everything and could not survive because COVID led to an economic crisis.

*Interviewer*: So, what do you do with all the free time now?

*Jingfan*: At least not so much gaming anymore. I used to play every day, but now I must take better control of my life. I need to spend my time on more meaningful things.

*Interviewer*: But you said you did play last week?

*Jingfan*: Well, I also feel anxious staying at home all the time. And gaming relieves my anxiety.

Gaming still serves a function for Jingfan, but mainly as an antidotal and instrumental activity. His gaming time is now down from an hour per day to two or three hours per week. Currently, those gaming sessions consist mainly of *Honor of Kings* (王者荣耀)—a mobile multiplayer battle arena game [note: this was played by almost all Chinese interviewees and survey respondents]. He has recently started using the so-called “game companion” service, which allows users to hire a stranger to play with. This provides Jingfan the feeling of not playing alone, but also does not require him to think about the feelings of his family or friends: he can be socially engaged, and his achievements are witnessed by others, but he is not “imprisoned.”

*Jingfan*: With the companion, we usually chat about the game, but more importantly, also about our personal lives, so they become also sort of mental companions for me.

*Interviewer*: Would you say the companion is more important than gaming?

*Jingfan*: No. I love the feeling of achievement, which I can have only by playing. For example, getting the level-ups or a Penta Kill [*beating five successive enemies*]—it’s this good feeling. I think gaming is mostly about these achievements.

The pandemic had affected Jingfan’s entire value system and made him rethink his priorities in life. This moved gaming into a paradoxical position: he feels anxious and guilty for having lost his job and being in financial trouble, but even though gaming helps him relieve this anxiety, his perception of gaming as “only entertainment” and no longer meaningful has led to less gaming. At the time of the interview, Jingfan had found a new part-time job he could do from home and was replacing his previous gaming time with movies and TV series. In other words, the diminishing of entertainment in Jingfan’s new value hierarchy was not unambiguous after all, but the successful regulation of gaming made him more confident about his new life goals.

### Increased gaming activity: Maria (#12), “stress or loneliness”

Maria is a middle-aged woman with a husband and pre-school children. She used to work in the health sector but has now moved to reeducate herself with new studies. They live in a smaller town, and according to her, the pandemic has worsened the local atmosphere. She feels that people are “hysterical.” For instance, “you cannot make even a small cough in the market, and if you do, they take notice of that and start rumors and then everyone avoids you.” Maria feels especially vulnerable due to her health sector background. People suspect the virus will eventually arrive to the town via those who have contacts with the health sector.

*Maria*: If the COVID app gives an alarm for someone, people say this person now has the virus. But that alarm does not mean you have it. It’s just a rumor. This town is not yet even listed by the officials as having had any cases.

*Interviewer*: How would you describe the current state, emotionally?

*Maria*: I would use the word “loneliness.” Different emotions arise in people, and this makes it difficult for us all. You may end up alone with your own emotions.

Maria’s children need more attention at home, as daycare services are not available every day. Gaming has been part of her life for a long time, also having met her husband at a gaming event: “We represent an older generation, and it was unusual for women to play at the time.” Maria used to play story-driven games on the PC and PlayStation, but that is no longer possible, as she does not have the time to immerse herself “properly.” Instead, she has mobile games as part of the daily routine. Although she considers the immersive features of mobile games inferior, she is strongly attached to them.

*Maria*: I am the kind of a player who commits to the videogame for a long time. For instance, this *Pokémon Masters*, which has daily energies and rewards, I always spend every resource and collect every reward that is available during the day. Every day. I’m very committed.

*Interviewer*: Can you tell us about your gaming, on a weekly scale?

*Maria*: Well, I like slow mornings, and a part of it is that I open my mobile games. Or, when daycare is available, the morning is of course a total mess—I need to get the kids ready to go in a hurry. But then I make coffee, take my slow morning, and go through all the gaming routines. I might play later throughout the day as well, but at least in the evening again.

In addition to the above, Maria uses *Zombies, Run!* (an exergame with an unfolding narrative) when walking the dog. This motivates her to walk longer, as otherwise the walk tends to fall short. At other times, easily accessible mobile games, which can be picked up at any time, fit her life routines and make life, in her own words, “meaningful.”

*Maria*: I often forget to recover properly from daily stress. Therefore, gaming is very important to me specifically by giving me a reason to, well, just “be.” I’m not the kind of person who would just do nothing. I need to feel that I’m doing something useful, and maybe gaming is a means to assure myself that I’m indeed useful.

In Maria’s current situation, stress and loneliness take turns, and it is sometimes unclear which one is present. She recovers from stress by playing, which allows her to “just be.” However, her extreme commitment can hardly be described as *just being*, but as she later adds, it rather allows her to keep *being useful*. Gaming makes Maria feel useful even beyond her numerous family and occupational responsibilities, and as long as she remains useful, the feelings of loneliness stay in control. Thus, loneliness seems to be an outcome of “empty time” when she is not occupied with *meaningful* tasks—or immersed in stories where meaningful tasks can be lived through vicariously.

*Maria*: They added “mini stories” to [*Pokémon Masters*] recently, and these stories make it much more interesting… I’ve played it for a year now.

*Interviewer*: What are your emotions when you play those stories?

*Maria*: Hmm. I would say “joy.” The joy of success, as I complete each small story. I know I am better than average players, as every time I complete the story, I receive the very highest prize. And the prize helps me further up, and I feel rewarded.

She highlights that gaming is important to her especially during “crises” (she uses the term loosely), as they allow her to “get out of the daily stuff and immerse in stories.” Maria knows that the stories will always end well, which boosts her belief in her own life, too, by “making me trust that everything will be ok for me as well.” Gaming is meaningful by making her feel useful when things are well and stable, but also as comfort and support during worse times. Whenever time allows, she also plays with her sister remotely—these are experiences that she cherishes especially during the pandemic—but everyday constraints do not allow it often anymore. Maria would *like* her gaming to be social, too, but her pragmatic reality does not make it possible.

## Discussion

In psychology, the lack of strong theories makes it difficult to explain how an activity like gaming interacts with people’s wellbeing and lives in general (e.g., Bowman, 2016; Orben, 2020). In this discussion, we offer phenomenologically sourced hypotheses that can help map out potential paths toward such theories (Fig. 2). In line with the premises of nonconfirmatory research, our work was not driven by a theory, but we do address some post hoc links between our work and theories. The eight subordinate themes and hypotheses have been organized by our RQ2 (decreased, unchanged, and increased gaming time).

### Gaming less

#### “Life has taught me a lesson”

In the previous literature, we found no indications of people having decreased their gaming activities during the pandemic; however, our survey suggested that a large part (18%) of the respondents belonged to this group, especially in China. Two out of our 20 interviewees represent this group, and their rationales for the decreased gaming time were somewhat similar. They both clearly enjoyed gaming—one having competed on a high level in esports and the other expressing that gaming helps lift him up from negative moods—but in both cases the pandemic “woke them up” for being more “responsible” in life due to unexpected financial difficulties, one of them explicitly saying that “**life has taught me a lesson**.” Although gaming was never expressed as a cause or contributor to their difficulties, gaming less was framed as “growing up,” which, in turn, the pandemic had triggered. In other words, gaming lacked meaning, or was associated with irresponsible adult life.

- **H1**: In a crisis like the pandemic, gaming (or media use) tends to decrease for those who find it lacking in meaning, and/or for whom other life areas increase in meaning.

The hypothesis is associated with the process of *internalization*, as behaviors become “integrated with the individual’s existing values” (Kelman, 1958: 53). In a cultural context, players may integrate gaming with their value systems, which often involves predefined cultural norms that possibly conflict with those of gaming. For instance, recent work on gaming-related wellbeing in India has suggested that norm incongruities between gaming and culture lead to dissonance reduction by self-stigmatization (Snodgrass et al., 2021). Our H1 is consistent with such cultural dissonance models yet provides an alternative pathway: instead of reducing dissonance by integrating a “disorder” stigma component to one’s identity, players to whom gaming is not highly meaningful will, contrarily, reduce their gaming and choose to pursue goals that are coherent with culturally imposed and/or other values.

### No gaming time change

Both the previous literature and our survey indicated a large part of people maintaining their gaming habits, in terms of hours played, during the pandemic. Nevertheless, predicting *how* gaming maintains its position in one person’s life but not the other remains unexplained. For instance, the respondents of Barr and Copeland-Stewart (2021) reported that gaming serves as a helpful coping strategy by providing them “escape,” “socialization” and “stress relief,” and Ellis et al. (2020) reported that their respondents considered gaming to be beneficial for their mental health during the pandemic. If gaming satisfies people’s basic needs (e.g., Rigby & Ryan, 2011), and the basic needs are interfered with by the pandemic, how come some people only maintain their old gaming habits? Seven interviewees said their gaming remained somewhat identical to pre-pandemic times.

#### “Peace of mind”

Three interviewees represent a subordinate theme characterized by a “**peace of mind**,” as one of these participants preferred to describe her current life. For them, gaming is a stable part of their identity and daily routines. Although the lockdown had a direct impact on their environment (e.g., switching to remote working), the pandemic did not significantly change their lives, and they felt the overall quality of their was now the same or even better than before. They had no reason to decrease or increase gaming hours, as they were already investing the “maximum” to the hobby they loved. The two things—not being affected by the pandemic and maintaining intensive gaming habits—might be linked simply by the fact that gaming (unlike sports, for example) did not change much during the pandemic.

- **H2**: In a crisis like the pandemic, the psychosocial wellbeing of those to whom an available activity (like gaming) that is a key part of their identity will be less negatively affected than those whose identity-relevant activities are not available.

This hypothesis follows the internalization premise of H1 and is consistent with research that indicates meaningful leisure activities to yield preventive, coping, and other wellbeing benefits when the activities are personally valuable (for a review, see Caldwell, 2005). Although people can be adaptive to life changes, attaching to new identity-relevant and meaningful leisure activities is typically a long process (Karhulahti, 2020; Stebbins, 2014), for which the availability of an existing, preferred activity should be critical for assessing the adverse effects of crises on wellbeing. It should be added, however, that not everyone has identity-relevant activities as part of their life and being one of those who do may simply reflect numerous stable resources (optimism, self-esteem, etc.) that contribute to psychosocial wellbeing (Taylor & Stanton, 2007).

#### “Annoyed but well”

Another three interviewees felt the pandemic had affected their lives negatively, but these feelings, especially due to longing for family or friends, were minor and they did not consider their lives to have changed much. For these people, gaming was also part of their routines, and they all played several hours daily. Unlike for those representing the “peace of mind” (to whom gaming was part of their identity per se), for these “**annoyed but well**” individuals continuous gaming served primarily and strongly a social function. In general, they enjoyed social interaction more, and this was also one of the main motivations for their gaming. Echoing previous findings on gaming as coping (e.g., Iacovides & Mekler, 2019), for these players, learned patterns of social gaming yielded accumulating benefits as a medium for belonginess and social support. Although they continued to play as they did before, the psychosocial returns were amplified in the new context.

- **H3**: In a crisis like the pandemic, the psychosocial wellbeing of those to whom gaming is a key part of their social life will be less negatively affected than those to whom gaming is not important.

In this hypothesis, gaming is not expected to automatically contribute to one’s social life. When assessing the relationship between gaming and psychosocial wellbeing, researchers should control the potential social aspects of play activities as well as the contexts in which they occur. The types of human social connection can differ radically by structure, function, and quality, which together represent the overall social effect on wellbeing (Holt-Lunstad, 2021). On the other hand, various types of social interactions may contribute to one’s social life; for instance, it has been recently suggested that even subtle interactions with strangers yield opportunities for social mindfulness that can contribute to short-term happiness (Van Lange & Columbus, 2021). Gaming may provide such opportunities, which we expect to serve protectively for psychosocial wellbeing in many ways (see Appendix 2).

#### “Coping with depressed feelings”

One of our interviewees had been affected by the pandemic more than others, and she was explicitly “**coping with depressed feelings.**” As a waitress, her work and daily social contacts were both shut down, and it was difficult for her to cope. She did not share a living space with her boyfriend or family (until recently), which contributed to her loneliness. In this situation, social gaming helped her to keep in contact with others, and it also brought her momentary feelings of achievement and success, following the distraction model of mood management (Reinecke et al., 2012). However, unlike those who were “annoyed but well,” for this individual gaming was not enough to shield her from the isolating effects of lockdown but she expressed symptoms of increasing depression and had lost interest in many daily activities. Gaming, among other technologies, was mainly a means for maintaining her few close social relationships—and utilizing gaming for coping thus limited by the scarce availability of her network. For her, neither online nor offline interactions provided enough resources for building sufficient resilience.

- **H4**: When social support networks are lacking, the coping potential of gaming (H2, H3) is limited and can easily be undermined by significant life setbacks.

This hypothesis continues with the premise of stable human resources such as optimism and self-esteem positively affecting psychosocial wellbeing (Taylor & Stanton, 2007), but moves further toward the psychiatric findings on resilience (for a review, see Herrman et al., 2011). For psychosocial wellbeing, resilience represents the individual’s capacity to maintain and regain homeostasis in the face of adverse life events. Numerous factors contribute to individual resilience, but in general, if the environmental stressors are significant enough without social support, even the most resilient individual will succumb to disorder or dysfunction. Accordingly, we expect gaming to yield limited wellbeing support, which can be countered by life setbacks that have significant adverse effects on mental health especially when lacking strong social support networks.

### Gaming more

In line with the previous literature and our survey, the most common change in people’s gaming habits during the pandemic was to play more. Out of our 20 interviewees, more than half (*n* = 11) indicated that their gaming time had increased. This increase, however, materialized in many ways depending on the specific context. Even though our study was not theory driven, previous evidence for a “rich get richer” model is worth citing here. Whereas some specifically stressed individuals may magnify their stress by increasing their gaming, a similar amount of gaming might lead to wellbeing benefits in balanced life situations (Snodgrass et al., 2014). Likewise, for some people, gaming seems to have a clear function as compensating for their offline social difficulties (Kowert et al., 2015), which nonetheless may require specific modes of gaming for this compensation to succeed (see Snodgrass et al., 2018).

#### “Calm and relaxed”

Somewhat similarly to those with a “peace of mind,” two of our participants were “**calm and relaxed**,” as they generally felt that the pandemic had had little effect on them. Even though some personally important events and opportunities had been cancelled, their overall life situation had been improved by the pandemic: one could now work remotely and no longer had to spend two hours on a daily commute, and another had new much-needed work projects due to her pedagogical skills being needed in remote teaching. Like those who were “annoyed but well,” these “calm and relaxed” individuals were motivated to play for social reasons. The pandemic had increased their gaming time due to the fewer opportunities to meet colleagues, family, and friends, that is, the activity compensated for their reduced face-to-face interactions.

- **H5**: For those to whom gaming is a key part of their social life, reduced offline social opportunities will be compensated for by increased social gaming.

This hypothesis continues from H3 and expects the previous subjective relevance of social gaming to significantly affect the degree to which gaming compensates for reduced offline social interactions. The idea that social relationships follow rules of substitution and satiation is relatively old in psychology (e.g., Baumeister & Leary, 1995), and at least one other study regarding gaming and wellbeing during the pandemic (Giardina et al., 2021a) has already suggested socially motivated players to continue gaming during self-isolation. Our hypothesis stresses the importance of subjective gaming history and habits when it comes to substitutional social response; namely, people who are already used to social gaming will be more inclined to utilize it, whereas for people with no or little experience the option is less likely.

#### “Stress or loneliness”

Five of our interviewees expressed “**stress or loneliness**” in a very similar fashion with those who were “annoyed but well.” Two of these five were mothers with school-age children, and the pandemic had affected their daily lives in many ways so that they expressed increased stress and occasional loneliness. The other three did not have children but noted that their daily activities and social interactions had been changed or limited by the pandemic, thus leading to similar feelings as with the above mothers. All these five interviewees played videogames daily and regularly, and a central motivation for it was to “relax” or “release stress” and compensate for the emerged social gap. To wit, gaming was a part of their daily routine already before the pandemic, but (like the “calm and relaxed”) the pandemic led them to play even more, as it helped them to compensate for the lost social events *and* to further manage the increased stress.

- **H6**: For those to whom gaming (or other available media use) is a key part of their identity, prolonged distress will be coped with by gaming.

This can be considered a sub-hypothesis for H2, which expected identity-relevant gaming to protect individuals from the potentially adverse wellbeing effects of pandemic-like crises. Although the hypothesis continues the well-known roles of gaming as coping (e.g., Jeong et al., 2019; Kardefelt‐Winther, 2017), our findings add to such models that gaming seems to serve as a functional coping mechanism primarily when it is identity relevant. The style and content of coping has been known to matter for individuals for a long time (Pearlin & Schooler, 1978), and mechanisms that are familiar are likely to be preferred. That said, we highlight that our hypothesis does not predict the outcomes for the coping; for instance, previous qualitative work with highly engaged players found such coping attempts occasionally problematic, providing only temporary relief without removing the source of distress (Shi et al., 2019).

#### “Combating setbacks”

Like the interviewee who was “coping with depressed feelings,” three individuals spoke explicitly about having started gaming or having radically changed their gaming habits to adjust to a difficult life situation. Whereas many previously discussed individuals had increased or changed their gaming only slightly (e.g., playing more socially), for these three individuals gaming was part of “**combating setbacks**” that had affected their lives significantly: the first had lost his job permanently, the second had her work fully suspended, and the third had lost her friend to the COVID-19 virus. For all of them, gaming had emerged as a coping mechanism, which helped them through an exceptionally difficult time—and the second participant eventually finding a new remote occupation in an online game, too. At least in the first two cases, gaming served generally as *escapism* (bidirectional) rather than *escape* (monodirectional), i.e., it allowed them to move away from the problems during the search for solutions (Giardina et al., 2021b), perhaps with a restorative coping effect (Reinecke et al., 2012). Unlike the participant who was “coping with depressed feelings,” none of these three had a partner but they did have flexible and strong social networks—gaming was chosen as their preferred subjective means of escapism, without conflicts or external influences from parents or peers. They described in detail how play provided them with various types of comfort, pleasure, and/or satisfaction.

- **H7**: In a personal crisis, subjectively initiated or increased gaming can support one’s psychosocial wellbeing if the activity does not cause conflicts in the person’s hierarchy of values.

This hypothesis highlights the importance of autonomy in gaming–wellbeing relationships. Namely, we expect that enforced or externally motivated gaming activities do not contribute to wellbeing in crises, but only when the activities derive from autonomous personal choice. The significance of autonomy is well recognized in the psychological literature. For instance, self-determination theory considers it as one of the basic psychological needs that contribute to human wellbeing (Ryan & Deci, 2017). Additionally, the hypothesis takes into consideration the potential value conflicts that can undermine the supportive role of autonomous gaming. Following H1 and H2, if gaming is chosen against the individual’s culturally (e.g., Dressler, 2022) and/or socially (e.g., Gregersen, 2018) defined value structures—for example, if gaming would conflict with valued occupational and social obligations—we expect the psychosocial wellbeing benefits of the autonomous activity to be significantly negated.

#### “Playing too much”

Finally, we had one interviewee who felt that he was “**playing too much**.” He expressed having had trouble controlling his gaming ever since childhood, and he explicitly used the term “addicted” when speaking of his gaming history. The interviewee stressed that he never plays with family or friends, but rather alone. Due to the pandemic, he lost his job as a clerk, which left him “bored” and filling his days with gaming as well as watching videos in TikTok and Baidu.

- **H8**: In strong personal setbacks, those with a long-term tendency to lose control over their gaming have an increased risk to do so with a downward spiral.

This hypothesis is consistent with theories of pathological behaviors, according to which related disorders are strongly associated with vulnerabilities, such as childhood disturbance (e.g., Brand et al., 2019; Orford, 2001; West & Brown, 2013). The hypothesis further utilizes the previously discussed patterns of gaming as a coping response (e.g., Shi et al., 2019). We expect individuals who are vulnerable to gaming-related health problems, such as active players with social anxiety and emotional regulation deficits (Benarous et al., 2019; Wichstrøm et al., 2019), to respond to strong personal setbacks by negative coping-by-gaming (see Hussain et al., 2021), which in turn can deactivate the problem solving of the faced setback and generate a downward spiral that hinders the attempt to overcome the setback.

We observed a great diversity of life contexts between the interviewees, which complicates the experiences of the individuals beyond what our limited synthesis can present. For instance, some of the interviewees *would have wanted* to increase their gaming, but the boundaries set by their current life situations (family, work, etc.) did not allow it. On the other hand, for some interviewees who kept gaming as much as before, the pandemic had changed *how* they play, for instance, by starting to provide in-game emotional support for others. Following the above, we further highlight the interviewees’ multiple ways of experiencing social interaction. Whereas many were explicitly motivated to play because they felt the need to be with others (remotely and/or physically), four interviewees felt that co-playing with family or friends would explicitly interfere with their personal space and make the experience stressful by forcing a demanding social etiquette. Considering the increasing understanding of the psychosocial role of gaming in people’s lives, future research should explicitly explore how different people *experience* sociality in gaming, and whether combinations of individual, sociocultural, and other differences (see Kaye et al., 2017) translate to phenomenological profiles where design components, such as social features, serve different roles (see Appendix 2).

## Limitations and Future Directions

Because the goal of this study was to investigate people’s lived experiences, we did not apply validated measures of health and wellbeing. On the other hand, many constructs such as hedonic and eudemonic wellbeing, which are commonly utilized in the psychosocial wellbeing research of media use (see Meier & Reinecke, 2020), remain difficult to measure reliably. In future studies, it would be useful to combine phenomenological analysis with clinical interviews. This combination would allow comparison of the experiences with participant-specific expert assessments. Second, because we aimed to refine the qualitative findings into testable hypothesis, many of the in-depth interpretations had to be streamlined, which makes our reporting slightly different from conventional IPA studies. Our Finnish data are available for open scientific reuse, so we encourage scholars to reanalyze the transcripts, which could further contribute to the psychological knowledge of gaming during crises. Third, although we followed the emerging recommendations of stating findings as testable statements to facilitate cumulative science (Hannun, 2021), more work is needed to seek optimal designs through which the hypotheses can be operationalized. Finally, we highlight that our study was not designed to investigate differences between populations. The study of phenomenological differences between cultures and other groups might yield other valuable hypotheses.

## Conclusions

This explorative study was set up to map out how gaming operates in the lives and wellbeing of those who have actively played videogames during the COVID-19 pandemic. Informed by a descriptive survey (*N* = 793) that indicated people in China and Finland have either decreased, maintained, or increased their gaming habits during the pandemic, we investigated how these habit changes were associated with the participants’ lived experiences. Based on 20 interviews with Chinese and Finnish participants, the study produced eight subordinate themes and hypotheses.

Our findings demonstrate how the general increase of pandemic-time gaming did not manifest in all player groups, but in some life contexts gaming activity rather decreased due to reformations in subjective meaning hierarchies and values (H1). The study provides further qualitative insight into the heterogeneity of technology use effects by showing how gaming-related identities (H2/H6), social habits (H3/H5), unexpected external events (H4/H8), and their networked interactions (H7) ultimately lead to great variation on individual levels. This variation surfaces also in so-called “social gaming,” which has been suggested as the core wellbeing contributor of multiplayer games. However, some participants also described their gaming experiences to lose meaning when others were present. Policies that promote socially supportive gaming during pandemic-like situations should consider including personally meaningful solitary play in their recommendations and highlighting context specificity over generalization. Finally, considering that almost all our data points suggesting experiences of decreasing gaming activity came from China, we stress the importance of culturally diverse samples in the study of global phenomena.

### Electronic supplementary material

Below is the link to the electronic supplementary material. Freeform preregistration 10.17605/OSF.IO/NVC9W

Supplementary file1 (PDF 75 KB) **Supplement 1**: Interview topic outline (in English)

Supplementary file2 (PDF 62 KB) **Supplement 2**: Assisting coding manual

Supplementary file3 (PDF 472 KB) **Supplement 3**: Interviewee summaries

## Data Availability

All survey and Finnish interview data are available for scientific reuse in Finnish Social Science Data Archive. We have no consent or permission to share the Chinese interview data. Individual URL for each dataset: http://urn.fi/urn:nbn:fi:fsd:T-FSD3545, http://urn.fi/urn:nbn:fi:fsd:T-FSD3547

## Appendix 1

An exploratory survey was initially developed in the Finnish language with 43 items related to gaming and everyday behaviors, including demographic items. Because the survey served to assist and contextualize our upcoming phenomenological work, we only report selected descriptive statistics here, as per transparency. The data and survey materials are openly shared for free scientific use and further inferential analyses in the Finnish Social Science Data Archive (FSD). In Finland, the survey was distributed in collaboration with two major local organizations, the state-owned General Broadcast and the leading gaming event organizer, Assembly. The organizations shared the link in their gaming-related networks, the Finnish respondents thus consisting of those who interact with gaming content via the former or the latter. In total, 152 (113 men) respondents completed the full survey. The survey was translated and back translated into English by our research team, after which a team member translated it into Chinese with external back translation. This version was then distributed with the help of Zhi Tian Qiu Shi Information Consulting to Chinese videogame players. In total, 641 Chinese respondents completed the survey (total *N* = 793).

Related to the present study, the survey implied people to have decreased (total 18%), maintained (total 36%), and increased (total 46%) their gaming time during the pandemic (Table 3). For this study, another relevant item was the open question “Have you noticed other changes regarding gaming, esports, or related media use due to the restrictions (COVID-19)?” The open question was optional and received less responses. We organized the responses (*n* = 399) thematically; the nature and number of themes was defined by fitting all responses to at least one larger theme by axial coding. The complete coding process was done first with the Finnish responses and confirmatory coding with the Chinese responses. Five themes were needed to cover all responses: “Social gaming increased,” “Gaming with different people,” “Playing different videogames,” “Using different media,” and “Cancellations, life issues” (Table 4). Informed by the survey, we made small changes to the interview.

**Table 3:** Selected descriptive statistics of the survey (*N* = 793)

	Finland (*n* = 152)		China (*n* = 641)	
In what way have the restrictions of movement and gathering (COVID-19 pandemic) affected your gaming?				
I play much less	0	0%	34	5.3%
I play less	4	2.6%	107	16.7%
No effect	93	61.2%	193	30.1%
I play more	43	28.3%	253	39.5%
I play much more	12	7.9%	54	8.4%
In what way have the restrictions of movement and gathering (COVID-19 pandemic) affected your affected your reading habits (novels, comics, nonfiction, other literature)?				
I read much less	2	1.3%	50	7.8%
I read less	3	2.0%	116	18.1%
No effect	111	73.0%	245	38.2%
I read more	34	22.4%	197	30.7%
I read much more	2	1.3%	33	5.2%
In what way have the restrictions of movement and gathering (COVID-19 pandemic) affected your TV watching?				
I watch much less	4	2.6%	30	4.7%
I watch less	5	3.3%	121	18.9%
No effect	110	72.4%	186	29.0%
I watch more	31	20.4%	244	38.1%
I watch much more	2	1.3%	60	9.3%
In what way have the restrictions of movement and gathering (COVID-19 pandemic) affected your physical activity?				
I am much less active	9	5.9%	65	10.2%
I am less active	39	25.7%	154	24.0%
No effect	66	43.4%	216	33.7%
I am more active	33	21.7	172	26.8%
I am much more active	5	3.3%	34	5.3%

**Table 4:** Themes of the responses (*n* = 399) to the open question “Have you noticed other changes regarding gaming, esports, or related media use due to the restrictions (COVID-19)?” in the survey

	Finland (*n* = 49)		China (*n* = 350)	
Social gaming increased	14	**Example**: “Spending more time with friends overall, as all my old friends are now online with me.” **Example**: “It’s easier to spend time with friends. As everyone is home, there’s no trouble scheduling.”	87	**Example**: “Compared to before COVID, I have more time to team up with my friends in the games.” **Example**: “Playing games now create more opportunities to meet new friends.”
Gaming with different people	7	**Example**: “Playing more with my family now.” **Example**: “My friends who didn’t play before play now, as the bars are closed in the evening.”	27	**Example**: “I play with different groups of my friends; by doing that, we have stronger bonds than before.” **Example**: “I play with both friends and colleagues. It is fun to try different games and strategies together.”
Playing different videogames	5	**Example**: “Relaxing and simple games are more appealing now, instead of deep narratives and fighting.” **Example**: “Finally time to play games that I didn’t have time before.”	102	**Example**: “With the recommendation of my friends, I started to play *Honor of Kings*.” **Example**: “I started to play games on *66RPG* (橙光游戏) and *Taobao Virtual Life* (淘宝人生).”
Using different media	9	**Example**: “Using Discord and live-streams now all the time.” **Example**: “Watching esports instead of sports; not interested in football with empty arenas.”	5	**Example**: “Since the COVID lockdown, esports have become my medium of online communication.” **Example**: “Instead of watching re-live, I watch live esports nowadays.”
Cancellations, life issues	12	**Example**: “Weekly board game session is now in Tabletop Simulator.” **Example**: “School takes much more time at home now. So I don’t have time to play anymore.”	129	**Example**: “I have more time to spend on reading and study.” **Example**: “My friends are not gamers, and they don’t spend time on esports (as much as I do) now.”

## Appendix 2

Although this study was not designed to map out social experiences, our double analysis produced several codes related to “social play,” which were exploratively collected into a 5-level taxonomy of social gaming experiences. We include these unexpected findings in the appendix, as they may be helpful for psychologists addressing social technology use.
