# Supplementary material for: Eight Hypotheses on Technology Use and Psychosocial Wellbeing: A Bicultural Phenomenological Study of Gaming during the COVID-19 Pandemic
Source: Curr Psychol. 2022 Aug 22:1–19. Online ahead of print. doi: 10.1007/s12144-022-03586-x (PMC9393080; doi:10.1007/s12144-022-03586-x)
Supplement: Supplementary file 1 — Supplementary file1 (PDF 75 KB) Supplement 1: Interview topic outline (in English) [file 12144_2022_3586_MOESM1_ESM.pdf]

The outline for the interview

The beginning: going through the data protection, the purpose of the study, possibility that the data will be saved anonymously for open use.

Yet: we do not directly inquire about sensitive issues such as health, but some of the questions may addressed topics that are unexpected.

Yet: possibility to ask questions before starting.

1. What kinds of feelings or thoughts has the COVID-19 pandemic and the current state of emergency raised? Follow-ups:

- If you had to choose one emotion to describe the effects of the pandemic, what would it be?
- Describe some concrete changes in your daily life?

2. Describe a normal (e.g. last) week regarding playing one day at a time. Follow-ups:

- Has the pandemic affected the regularity of the week in one way or another?
- Are these the same games as earlier?
- Do you feel or think about gaming when not playing, or do you recall experiences afterwards?

3. Tell as accurately as possible about one of your gaming experiences during the last month. An experience that has specifically stayed in your mind. Follow-ups:

- What would be a describing *feeling* of this gaming experience?
- Describe the 'highlight' in this experience?
- Describe the 'lowlight'?

4. **If you are playing with friends or family, describe how you think they felt during the experience.** (If you are gaming alone, think of someone you know who plays.) Follow-ups:

- Do you think that the pandemic has affected these experiences?
- Tell us about the dynamics of the gaming experience, whether alone or shared?
- Imagine a person playing this game. Why and what they would like?

5. Choose some other thing you like to do in your pastime. Tell, as before, a) the weekly schedule and b) as accurately as possible about one of those experiences during the last month.

6. Choose an important thing that you have not been able to do during the pandemic, or something that has been more difficult to do. Describe it.

- Describe similar events in the past.

Thank you!
