# Supplementary material for: Eight Hypotheses on Technology Use and Psychosocial Wellbeing: A Bicultural Phenomenological Study of Gaming during the COVID-19 Pandemic
Source: Curr Psychol. 2022 Aug 22:1–19. Online ahead of print. doi: 10.1007/s12144-022-03586-x (PMC9393080; doi:10.1007/s12144-022-03586-x)
Supplement: Supplementary file 2 — Supplementary file2 (PDF 62 KB) Supplement 2: Assisting coding manual [file 12144_2022_3586_MOESM2_ESM.pdf]

Supplement 2

An assisting manual of contextual (not directly phenomenological) patterns, based on the first six interviews (3 + 3) and revised after the rest of the interviews (7 + 7). The coded patterns were not assessed quantitively, but they supported the comparative phenomenological analysis of individual experiences.

1. *Social*

1.1. *Social Play*

"Experiences where other people are involved in the participant's gaming or play. Also includes instances when other people are not playing."

1.2. *Everyday sociality*

"Daily or weekly experiences where other people are involved in the interviewees' lives actively or passively."

1.3. *Absence of sociality*

"Experiences where people are alone or not social. For instance, forbidden to participate in social activities due to COVID-19 restrictions, which influence the relationships with families, friends, colleagues, and strangers."

2. *Emotions*

2.1. *Everyday emotions*

"States of feeling in daily life. For instance, responses to events that happened."

2.2. *Emotions in activities*

2.2.1. *Achievement or completion*

"Satisfaction or other emotions related to getting things completed or achieved."

2.2.2. *Absorption* or *immersion*

"A state of flow, mesmerized, or deeply focused on oneself in play. Also applies to non-gaming activities."

2.2.3. *Development*

"A description of players' efforts to make progress in activities/gaming or reflecting on it."

3. *Life during Covid*

3.1. *Routines*

"Regular procedures, tasks and duties to be done on a daily/weekly basis especially during the pandemic. Also includes non-COVID-19 related routines."

3.2. *Time allocation*

"Distributing a certain amount of time on a specific activity during the lockdown."

3.3. *Practical matters*

"Difficulties and changes in people's lives during the lockdown. For instance, unemployment, financial crisis, restrictive regulations, and cancelled/postponed social activities."
