# Supplementary material for: Eight Hypotheses on Technology Use and Psychosocial Wellbeing: A Bicultural Phenomenological Study of Gaming during the COVID-19 Pandemic
Source: Curr Psychol. 2022 Aug 22:1–19. Online ahead of print. doi: 10.1007/s12144-022-03586-x (PMC9393080; doi:10.1007/s12144-022-03586-x)
Supplement: Supplementary file 3 — Supplementary file3 (PDF 472 KB) Supplement 3: Interviewee summaries [file 12144_2022_3586_MOESM3_ESM.pdf]

Supplement 3:

Summaries of all interviewees (n=20).

#1 Chinese Man

Pandemic

Lost his job as a manager. Life during lockdown was emotionally difficult.

Spent all free time on gaming

For several months, played to feel good and meet people. Gaming was both competitive and social. Served as an escape and to manage the pressures of the life situation.

Found a new job online

In the new job, he talks with people daily and learns about their struggles with the pandemic. This has made him see his own life in a more positive light.

The interviewee struggled at home during the lockdown after losing his job as a representative manager. After that, he found a new job in the broadcast sector, reading audience letters and offering comfort and advice, as well as playing music. In this work, he receives letters about loneliness and relationships. In free time, he enjoys singing and dubbing as a part-time vocation. He spent approximately 6-7 hours dubbing per week. His new work made him see that the pandemic was heavily affecting people's moods, and they were desperately seeking ways to vent their emotions. He felt that during the pandemic his audience had a lot of dependence on his broadcase. Sometimes it was challenging for him to remain positive. As a gamer, he played PUBG (Player Unknown's Battlegrounds) and Honor of Kings, and especially the former. After he became unemployed, he spent almost all free time playing games to relieve pressure and to avoid problems in daily life. He also met a girl, as a potential romantic partner, but that relationship ended after two months. In general, he felt challenged by the life situation, and it did not help that his travel plans were entirely cancelled due to the restrictions and financial issues (unemployment).

#2 Chinese Woman

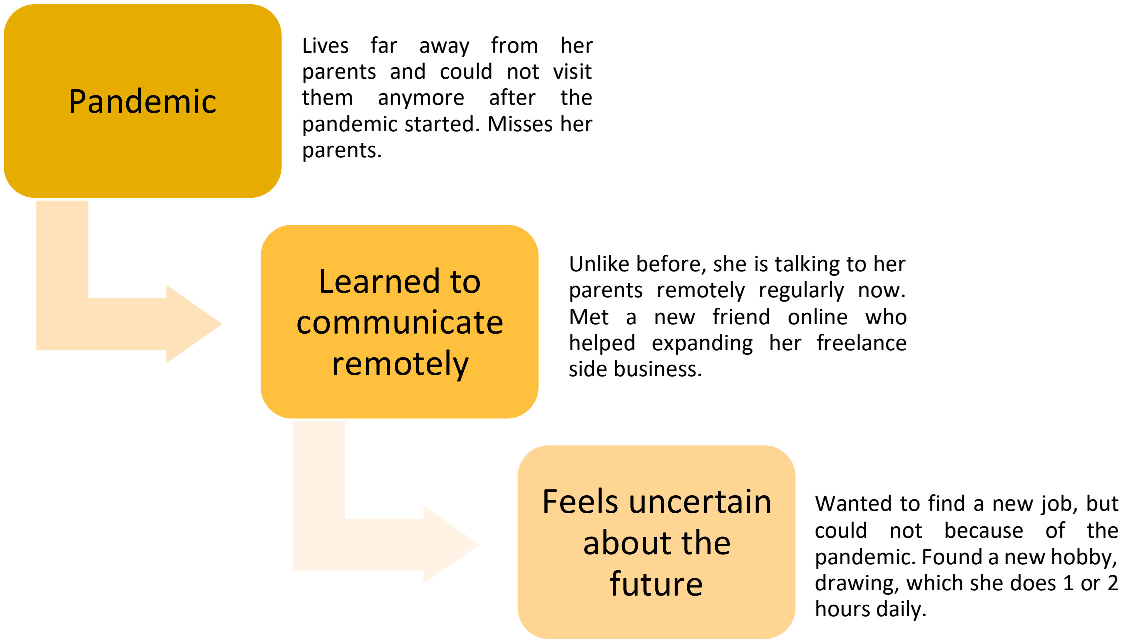

The interviewee works as a manager assistant. During the lockdown, she missed her parents back in her hometown. She was supposed to change jobs, but those plans were postponed. She hopes to get a new job soon. In her company, COVID-19 led to many projects being placed on hold, and her paychecks were slow in coming. However, the pandemic did increase her remote social connections with friends and family. As a self-identified gamer, she plays Honor of Kings every day and used to play Mahjong with her family, too. In Honor of Kings, she prefers to have "skilled" friends with her to play on a high level, but she also enjoys meeting new people via gaming occasionally. During the pandemic, she met a student who became a good new friend outside the gaming world, and a man who helped her expand her freelance side businesses within his circle of contacts. In her free time, she spends ten hours a week engaged in drawing, which she did not do before the pandemic. She used to meet friends from other provinces physically, but now she had to cancel due to the restrictions caused by the pandemic.

#3 Chinese Man

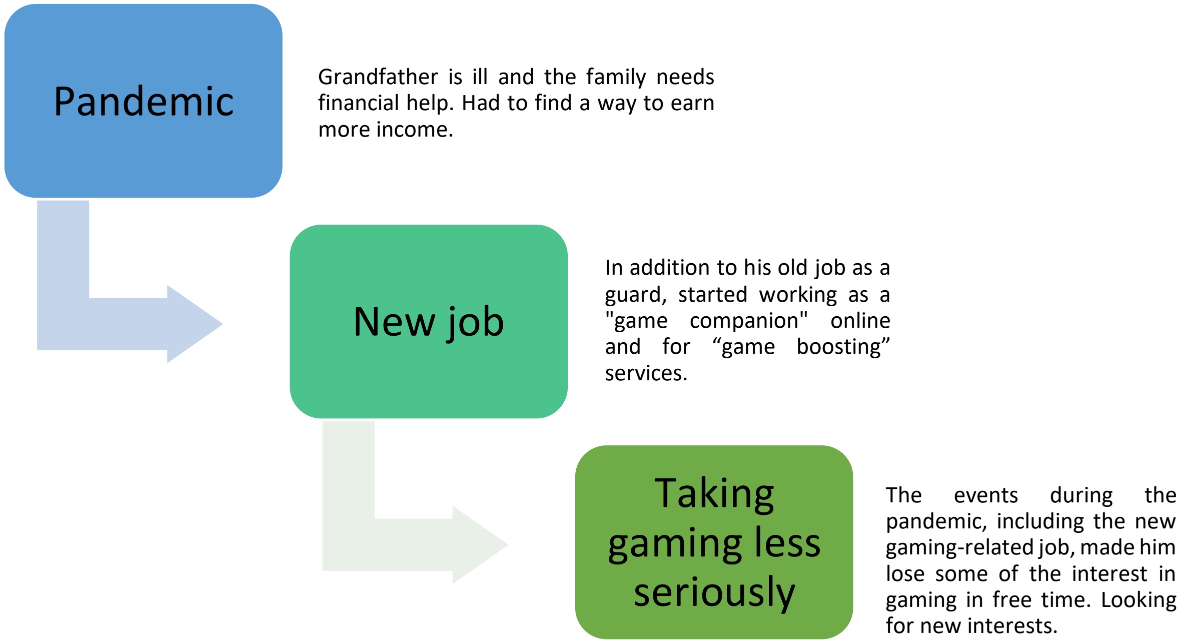

The participant’s parents lost their jobs at the start of the pandemic, and his grandfather fell sick. He took a job as a security guard and became a part-time “game companion” and “game booster” to support his family. He also sold his online game accounts to survive a personal economic crisis. As a game companion and booster with more than 50 clients per week, he met many kinds of people from “sweet couples” and fascinating individuals to not-so-nice customers (who did not pay). He mostly plays Honor of Kings, PUBG and QQ Speed. While playing with clients, his job is often to lead them to win. In the case of failure, he would soothe the client verbally, serving five or six people every evening. He used to play all his free time previously—enjoying both social and solitary play—but the family crisis and the new job made him lose a large part of his previous interest in gaming and look for other interests, such as running and spending time with friends and family. He has also met a girl with whom he fell in love, but he does not dare to tell her about his feelings. In general, his social life has been enriched during the pandemic due to his active participation in online interaction, which takes place in gaming environments only partly.

#4 Chinese Woman

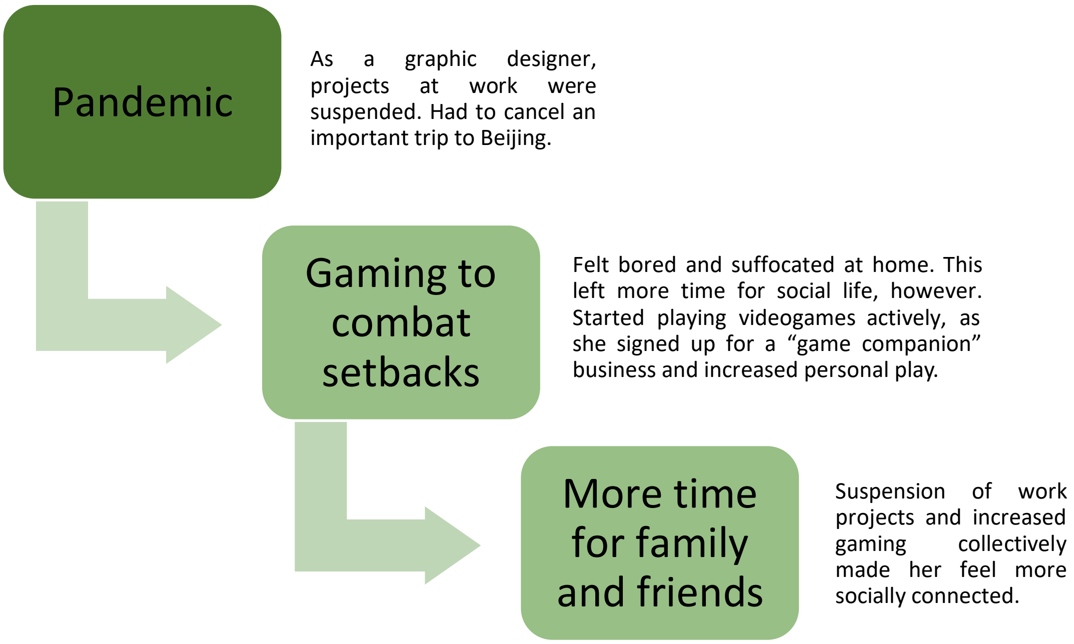

The interviewee works as a graphic designer and enjoys playing snooker every two to three days. Her plan to attend an important show in Beijing with her friend was canceled. During the lockdown, she feels bored and smothered, as her company's projects were suspended and her income was frozen. She decided not to quit her job but wait, nonetheless. The lockdown and work suspension helped her to reconnect with friends, however—like Interviewee #3, she started a "game companion" business to compensate for the frozen income. Her clients pay her to provide a better playing experience and she would sometimes provide guidance regarding the played videogames. Being a game companion gave her a chance to meet new people, which permitted emotional interactions, and an opportunity to make the customers happy. Before the pandemic, she played only once a month, but now she plays every day. As for her personal preferences, she enjoys playing QQ Speed and Honor of Kings. In her free time, she prefers to play alone without needing to think about others' feelings. On the other hand, she uses other remote media to socialize with her close ones.

#5 Chinese Woman

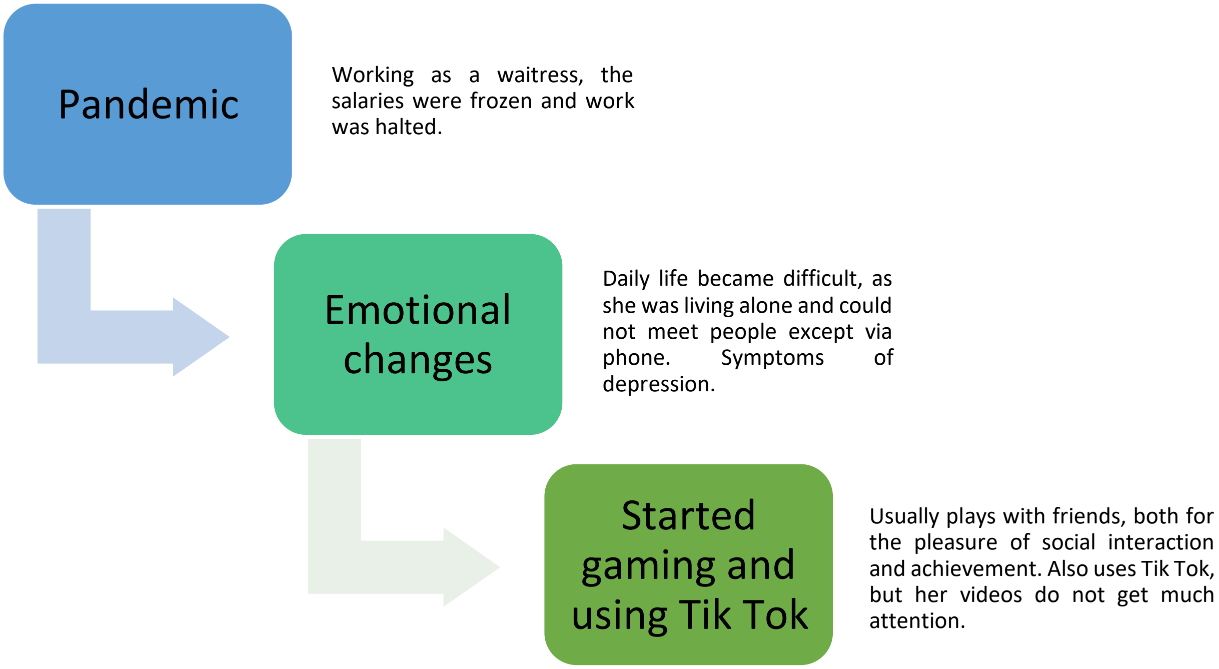

[Some key parts of this interview have been removed to protect the privacy of the interviewee.] The interviewee used to work as a waitress. During the lockdown, her plans to travel with her boyfriend were put on hold, and to make matters worse, her employer did not pay her salary on time. Living alone with scarce social interaction made her feel depressed and lonely. She used to play PUBG, but her account was deleted. After that, she moved to play Honor of Kings and now plays it daily. She liked to interact with her friends and considers gaming as an effective means for communicating with them, allowing talks about daily life and gaming strategies. Gaming and social media are tools for her to survive the pandemic but is not easy.

#6 Chinese Man

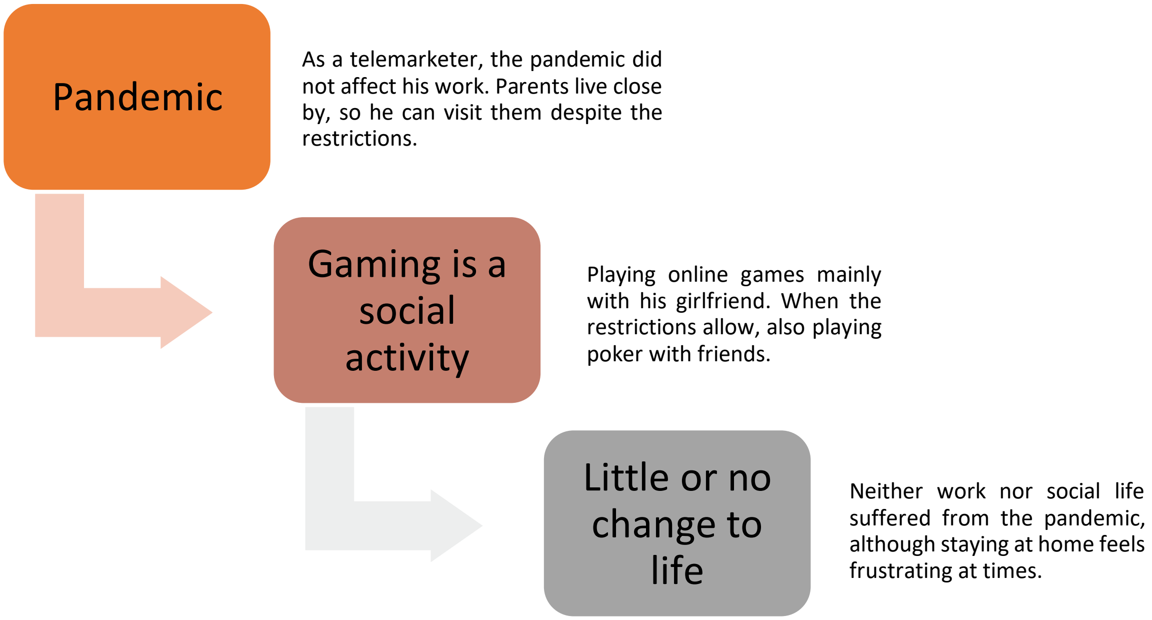

The interviewee works as a telemarketer. The lockdown did not significantly affect his social life, as his parents live in the same area and his girlfriend with him. He is proud of how the Chinese people have handled the COVID-19 situation together, but also a bit frustrated for the quarantine limits. Most of his leisure activity involves social interaction, such as enjoying beers with his friends and learning about Chinese poker traditions (Dou Di Zhu and Zha Jin Hua). He plays Honor of Kings daily too, but mainly to connect with his girlfriend and now (sometimes) also to kill time during the quarantine. He consciously controls play time so that gaming does not take all his free time. He is a big fan of Jay Chou, as well as the music of Wubai and Yu Zhang, which are important to him, and he spends some of the time every day to immerse himself with the artists.

#7 Chinese Woman

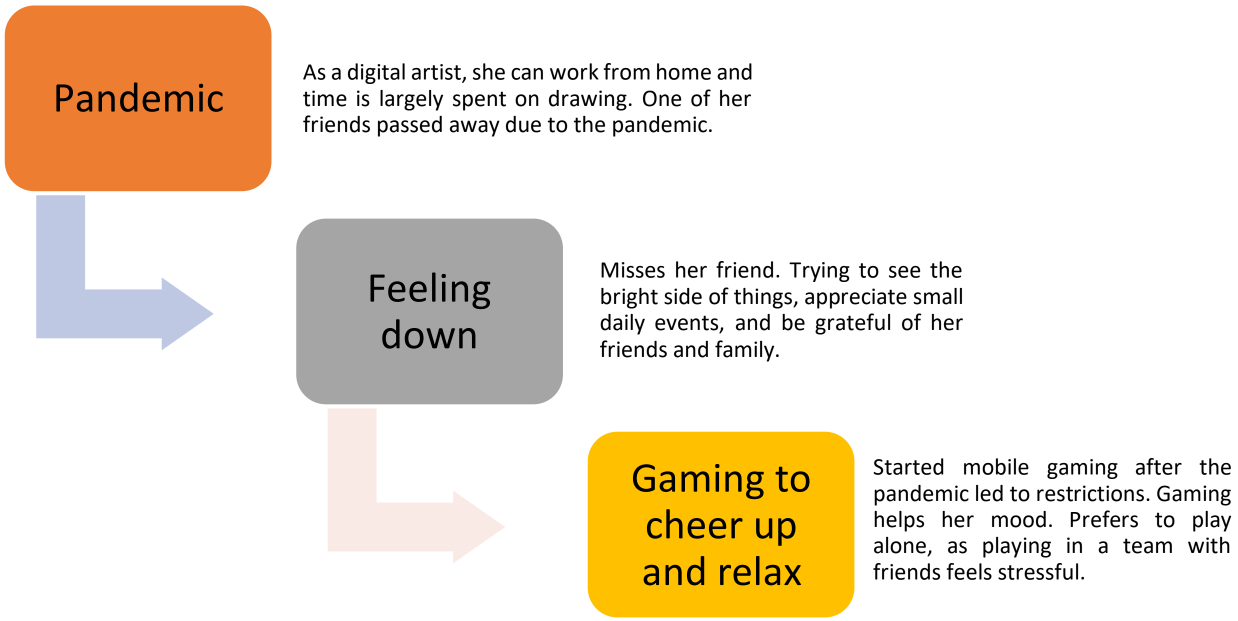

The interviewee works as a digital artist, and thus the pandemic did not affect her daily routines much—she still draws seven hours per day, which she always does by listening to music at the same time. However, she lost a good friend to the COVID-19 virus, due to which her current emotional state is downhearted. She looked for ways to improve her mood and get over the sorrow; gaming was one of these ways. She has played a lot of different videogames over the years, such as Sword Heroes Fate 3 and Love and Producer, however, now she plays mainly Honor of Kings. She sometimes has negative experiences with strangers while playing the game, being verbally abused because of her gender, and she believes voice communication facilitates such toxic behaviors. Still, she prefers to play with strangers instead of friends, as she feels only then she can be fully herself and enjoy gaming in her own way. Watching documentaries and reading romance novels work for her in the same way. [Note: it is not clear whether her gaming has increased during the pandemic or not.]

#8 Chinese Woman

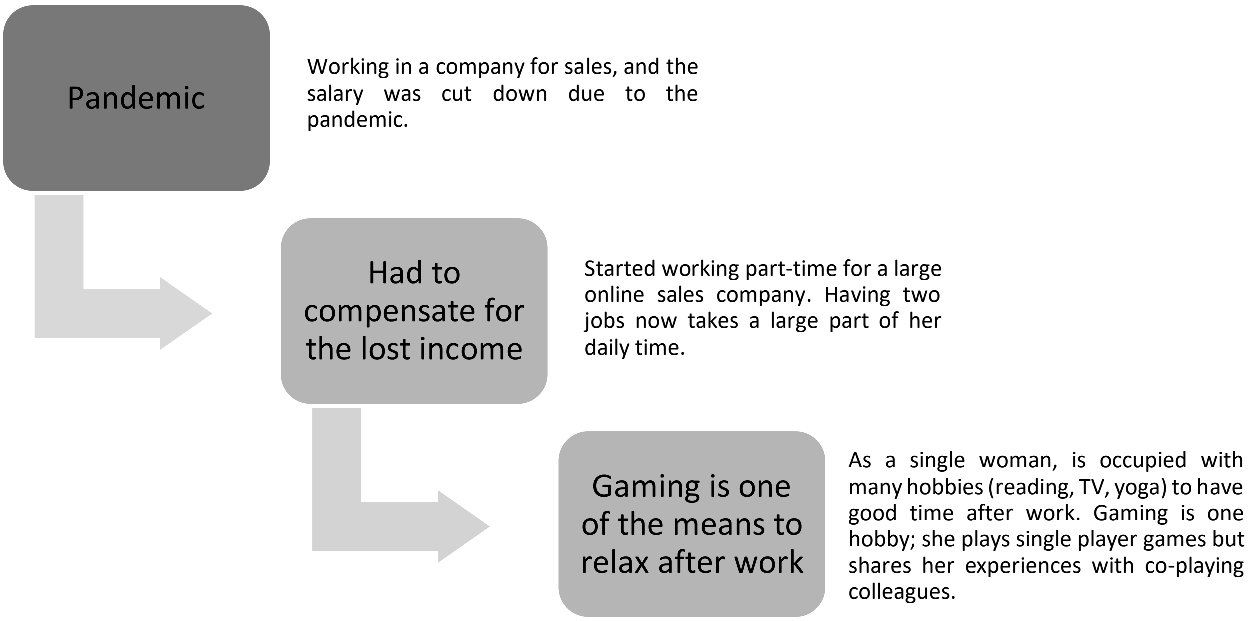

The interviewee kept her job during the COVID-19 lockdown, but the restrictive period heavily impacted her life, as her salary was cut down dramatically. She started a part-time job as a Taobao seller, specializing in selling clothes. Her plan of visiting her parents during the Spring Festival had to be cancelled to follow the Beijing government's request for citizens to spend the new year locally and limit traveling. Her main gaming titles are Love & Producer and Love Nikki, which offered her special cards and pops to celebrate the remote Spring Festival. She plays every day after work hours and shares her gaming experiences with colleagues. The parasocial relationships with videogame characters are important to her. She also developed an interest in yoga during the pandemic. At other times, she also enjoys music, Korean TV dramas, and family shows.

#9 Chinese Man

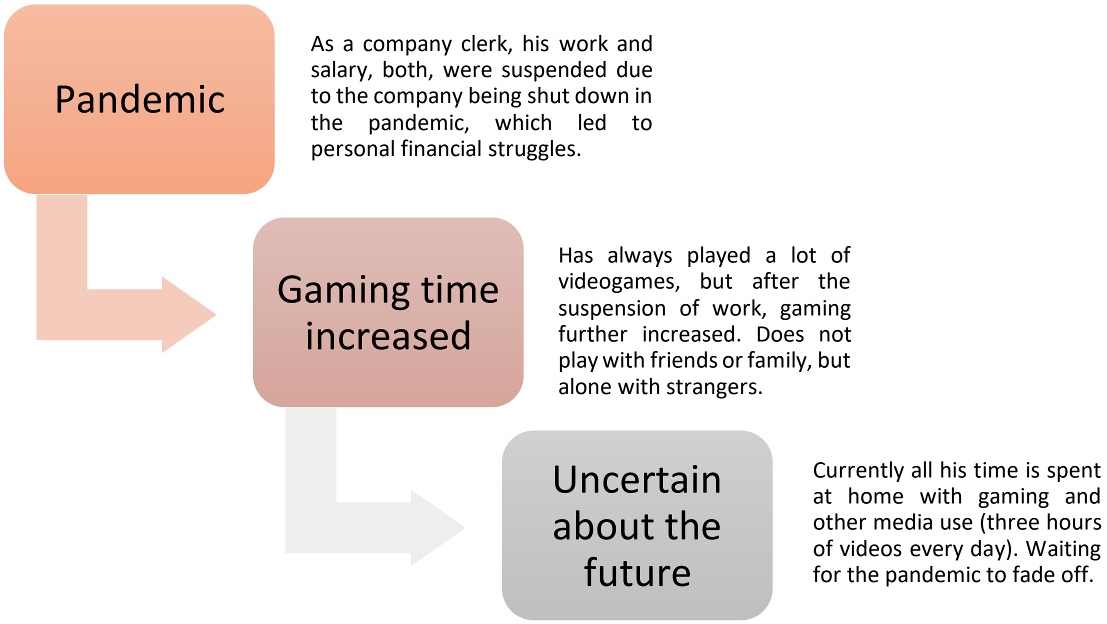

The interviewee works as a clerk in a small company, which was shut down during the pandemic. His work and paychecks seized, which greatly influenced his daily living and left him with plenty of spare time unpaid bills. During the lockdown, he has been unable to visit his parents. On the other hand, he self-identifies as “addicted” to gaming, as daily play takes continuously priority over other life issues. He used to play World of Warcraft (deleted the account to control his play) and more recently Crossfire, but now mostly Honor of Kings. He tends to get lost in the game while playing and feels that it represents an easy escape from personal problems, such as the financial struggle. He never played with family or friends, only with strangers, even though some of these players are rude and show him little kindness or respect. He also reads books, watches Tik Tok videos and surfs in Baidu several hours hours every day.

#10 Chinese Man

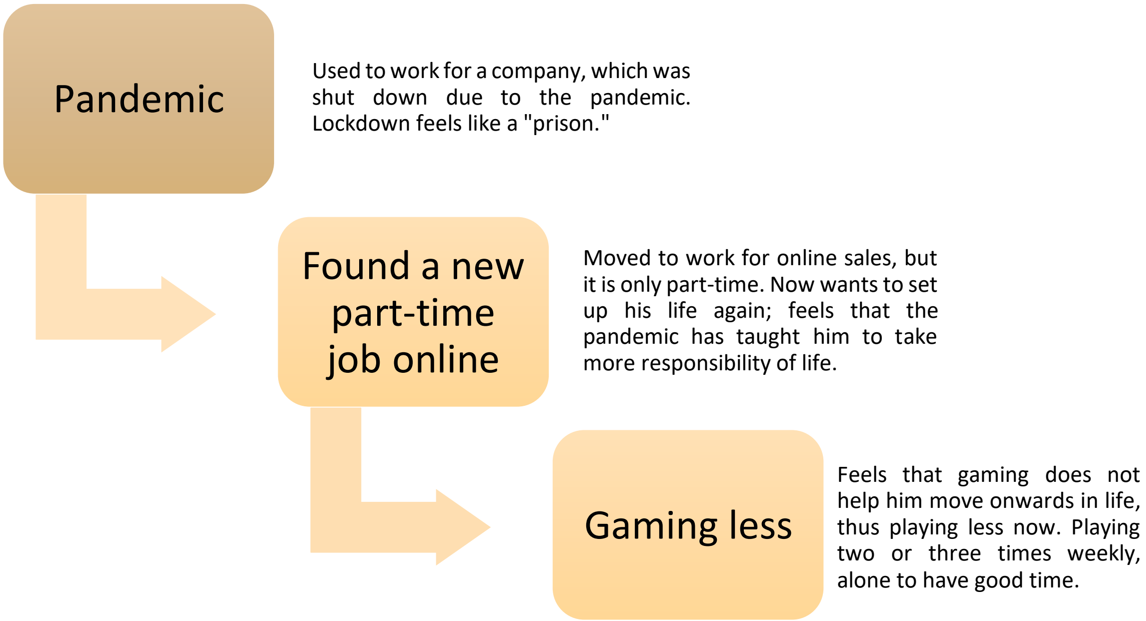

The interviewee lost his job due to a company shutdown during the pandemic. He found a new part-time job at online sales but describes the lockdown as “being in a prison”—he does not go out much yet feels locked because he *cannot* do that anymore. He has been forced to postpone the visits to his parents. He used to be an avid gamer, but during the lockdown realized the importance of practical life management, such as finances, which led him to move away from videogames. He considers gaming nothing but entertainment and now tries to spend as little time as possible with videogames. He plays Honor of Kings with strangers and likes to chat with others about daily life during play (sometimes using “game companion” services). He never plays with family or friends, because it forces him to take into consideration of others’ feelings, which interferes with his own experience. Instead, he is trying to have “real” social interactions with people. He also enjoys watching movies and dramas daily, which partially compensate for the reduced gaming time.

#11 Finnish Woman

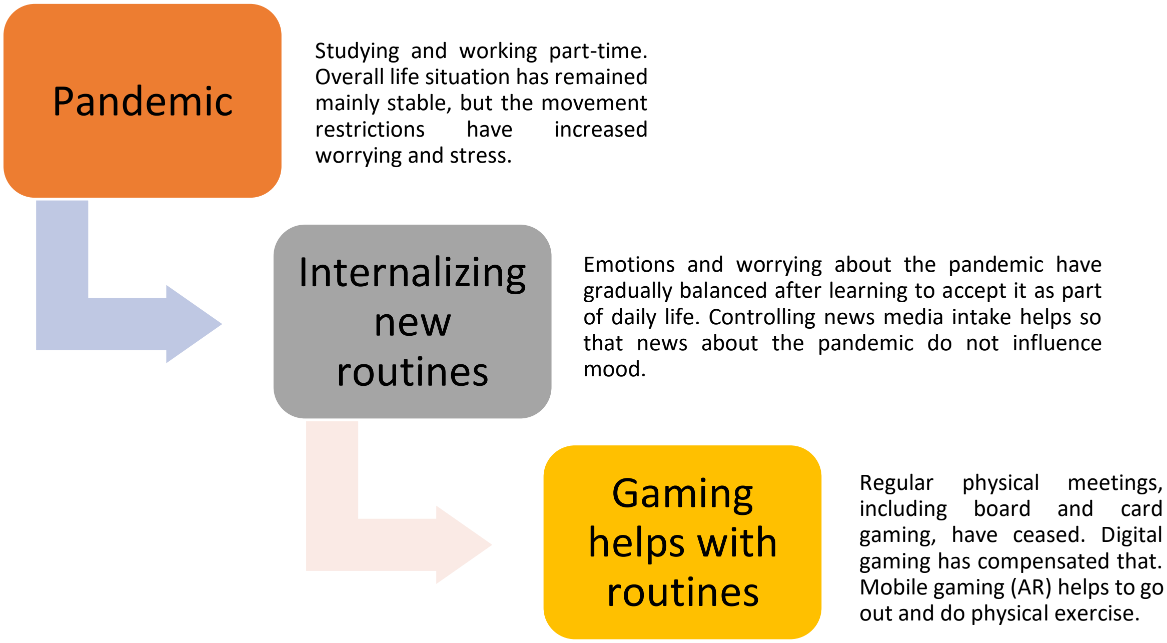

The pandemic had made the participant feel more stress and be more cautious about the future. She had restricted her movements outside, but is worried about the acts of other people, also wondering if it is safe to (have) spend time with other people. She has been gradually accepting the situation with less stress. Lessening the consumption of news media has helped her to ease these feelings as well as getting used to new routines and health/hygiene practices (such as using hand sanitizers) but she was still very aware of the virus. She feels that the pandemic has increased her time spent with videogames. She has stopped playing board games during the pandemic, as it is not possible to gather physically. She mentions a memorable moment from a Harry Potter AR game, which helps her go out—she feels excitement in battle scenes, during which her pulse got faster. Besides gaming, she likes to watch Netflix series with her partner. During the pandemic, most of the shared time with her companion had been during weekends.

#12 Finnish Woman

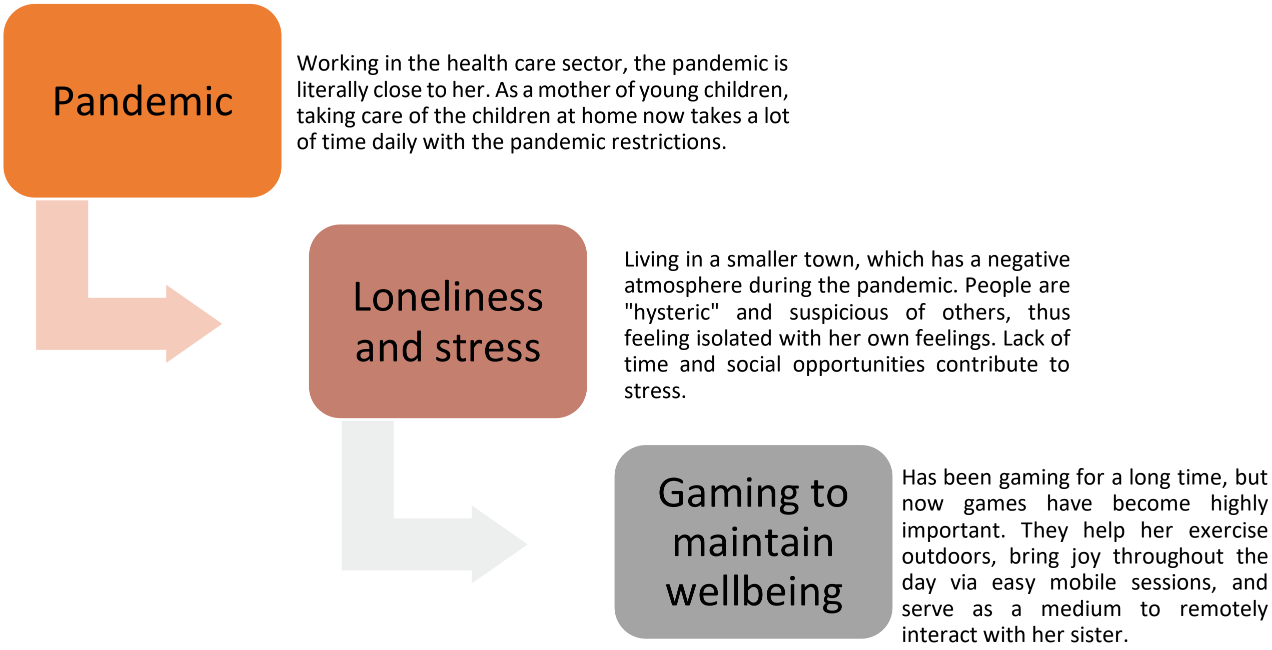

The interviewee is an extroverted mother of small children. She has a history of working in the health care sector, and currently works on new studies. She feels that the pandemic has been straining and affects the atmosphere of her hometown negatively. The COVID-19 virus stresses people and because of that people cannot support each other emotionally. The lack of social activities is hard for her. The interviewee plays mostly mobile games, as they required less time and attention than console games. She used to play console games before having children, and she sometimes misses playing them. Because of the pandemic, her children had been more at home, and she has less time for gaming. Mobile games provide relaxation and motivation to exercise (Zombies Run!). When she plays with her family, the social aspect and her children's experiences are a priority. She feels joy in videogame storylines and completion. For her, the importance of play has grown during the pandemic: videogames provide her with a chance to escape reality in a positive way. Playing multiplayer games with her sister, who lives far away, had increased. Besides gaming she likes to watch “easy entertainment” from the TV.

#13 Finnish Man

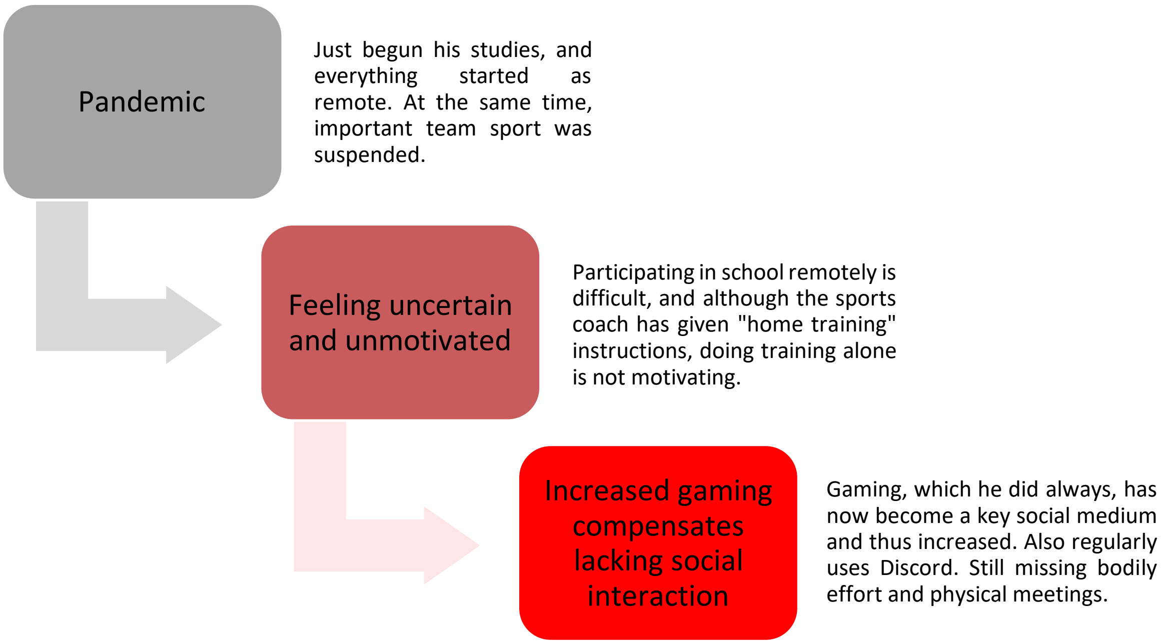

The participant had recently started his studies remotely. Student events and team sports activities are now cancelled due to the pandemic. The extra effort required to keep in contact with his team and his feelings of lack of group dynamics have caused his spare time to feel unfulfilling and unmotivating, and increased the time spent on solitary activities. He meets his friends less and the occasional participation in small social gatherings evoked feelings of worry and guilt. He feels that everyday routines and structures have disintegrated, and life is become unpredictable. He feels uncertainty about the future. Social inactivity is somewhat compensated through multiplayer games with teammates and communication with other students via Teams and Telegram, although the latter is strictly about schoolwork. The interviewee plays videogames on several days per week for a few hours at a time, which is more than before COVID-19, although starting his studies had also altered his schedule. In particular, he plays more social and multiplayer games than before. Experimenting and planning in videogames brought him joy and excitement that he no longer could easily acquire in ”the real outdoors.” The interviewee feels that conversing with others during gaming is more limited than in live situations.

#14 Finnish Woman

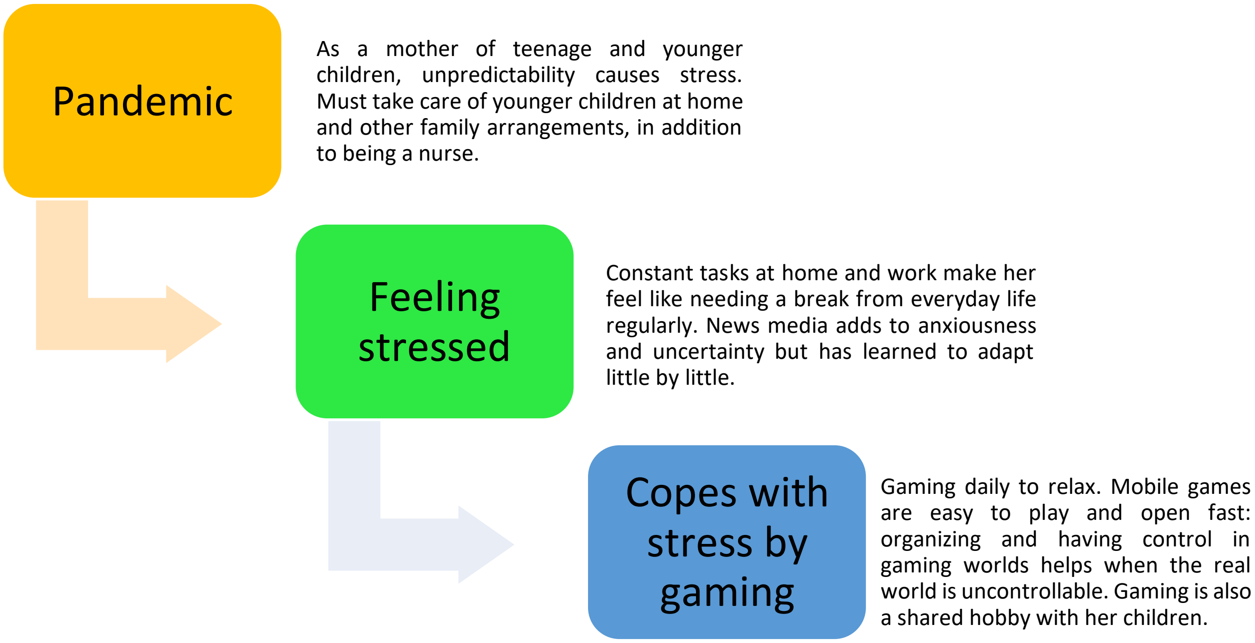

The interviewee is a mother of three children, one teenage and others much younger. She works in health care. Continuous media reports about the pandemic make her anxious, but she feels increasingly well adapted to the practices and restrictions set during the pandemic. The unpredictability of the pandemic, virus, medication, and vaccine development stress her. She feelt anxiety about COVID-19-related news. The interviewee plays mostly mobile games because they are easy to access on her phone. Previously she enjoyed other types of videogames frequently. Organizing things in videogames and aesthetically pleasing virtual environments relax her and give her a break from everyday life. She plays more mobile games since the beginning of the pandemic because of increased stress. The interviewee plays both digital and board games with her family, too, as a shared hobby and a way to connect and share experiences with her children. Watching movies and TV-shows have a similar function. The interviewee does not feel that family gaming has been affected by the pandemic, however, she is missing out on travelling and live music events because of it.

#15 Finnish Woman

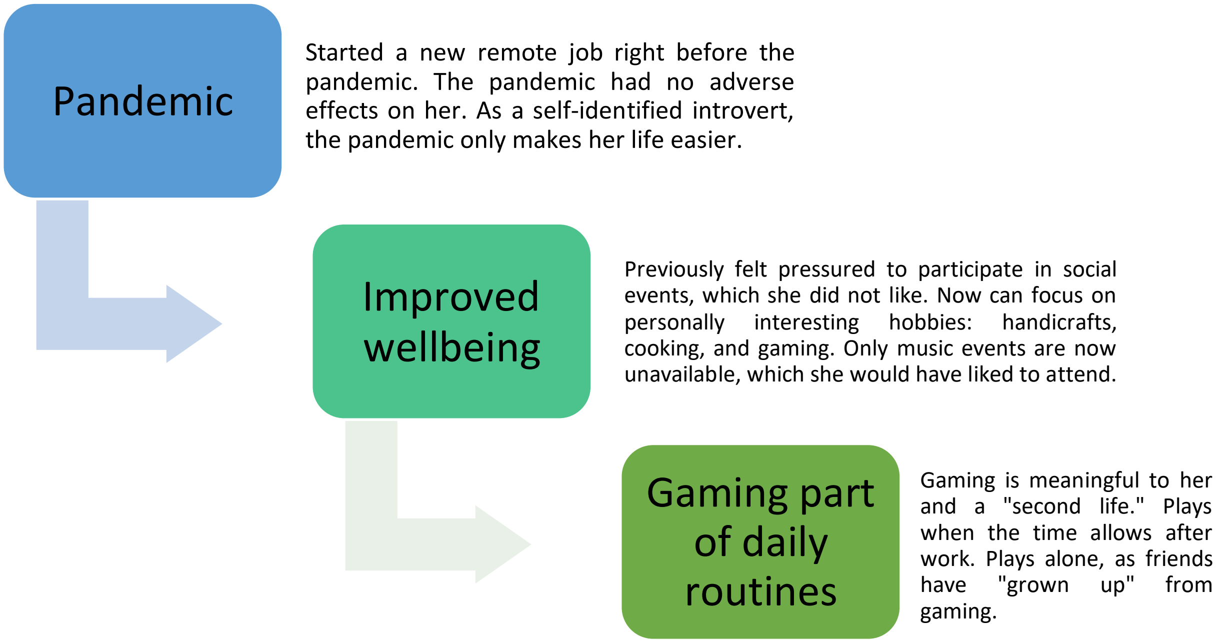

The interviewee describes herself as introverted, due to which she had enjoyed life during the pandemic. No longer did she feel any more pressure to participate in social events. She started at her job in the beginning of the pandemic and could work from home. She feels free and has more time and energy to spend on her own hobbies and interests. The interviewee spends her increased spare time on acquiring new skills and on hobbies. She plays videogames at night after work, and her gaming routines have evolved around her work routines. The interviewee had started playing videogames as a child and had strong emotional bonds around certain games. Especially important were memories of playing with her late father and teenage best friend. Music often triggered in her the desire to play a certain videogame. She experiences the emotional and aesthetic aspects of gaming strongly. Her friends no longer play videogames as they have “grown up” and started families. She does not like to play videogames with strangers and thus prefers to play alone. The only thing that she misses during the pandemic is live music concerts, as she feels that attending remote concerts does not provide a similar feeling.

#16 Finnish Woman

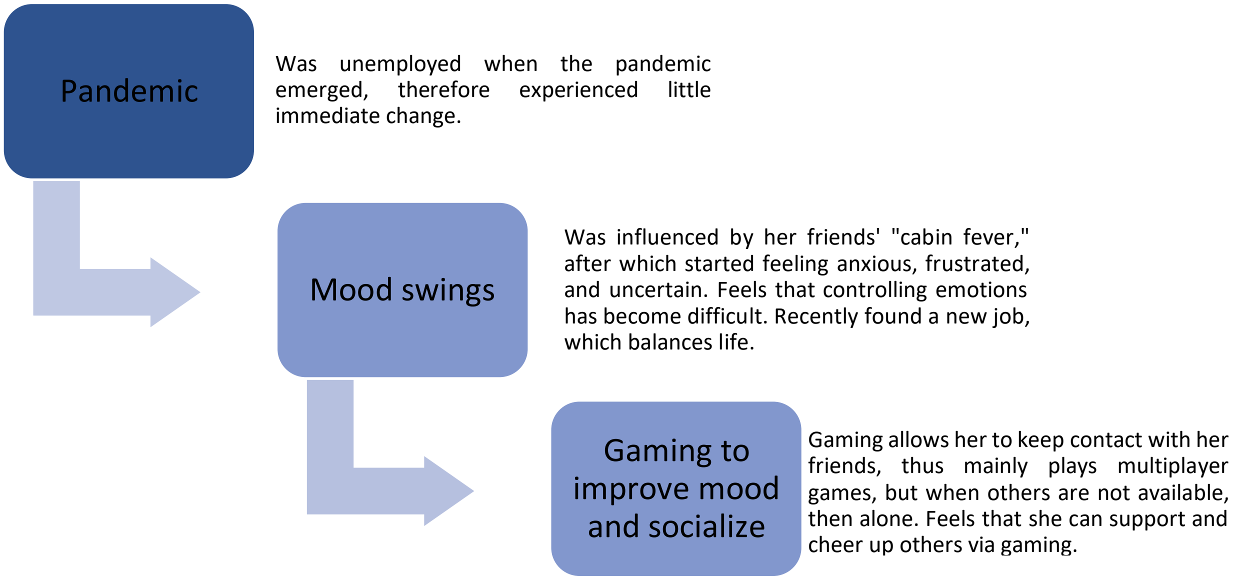

The interviewee was unemployed at the beginning of the pandemic, and thus feels that the pandemic did not affect her life immediately. Only after some time and their friends getting “cabin fever” she also started to feel negative emotions such as anxiety and uncertainty. The interviewee describes how both their negative and positive emotions became more extreme and more difficult to control. She started to feel forced to stay indoors as everything shut down, spending much more time on the computer. Communication with friends turned completely virtual. The interviewee plays videogames nearly every day and it has been a hobby for a long time. She plays more social and multiplayer games since the beginning of the pandemic to socialise, and videogame choices are made collectively within the friends group. Currently, the interviewee plays more multiplayer games than normally also because of temporary device issues. The interviewee feels she can “take care of” and support her friends by playing and communicating positively. The group plays varied videogames and compromises more during the pandemic, as the emphasis is more on the socialising and shared enjoyment than before. The interviewee knits to relieve stress and did it more in the early days of the pandemic. She misses attending social events such as Assembly with her friends and feels like the virtual Assembly would only highlight not being able to experience the real thing.

#17 Finnish Woman

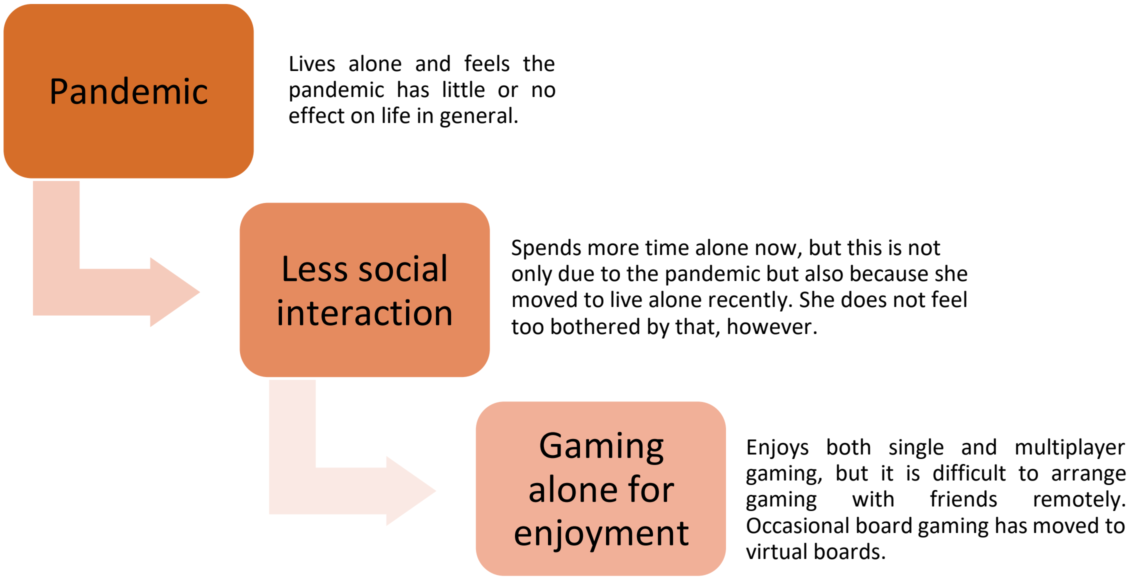

The pandemic has had little effect on the interviewee, who is a student. She feels that the pandemic has affected only her spontaneous activities and social life, i.e. not being able to meet with friends and relatives. As an example, the interviewee mentions playing board games: her previous weekly board game sessions have been replaced with remote sessions, but only once in 2-6 months. Despite that, she feels generally satisfied and content. The interviewee plays videogames daily for a couple of hours. She plays single player games more, as it is difficult to get enough people to join a multiplayer game and she dislikes playing with complete strangers. The social aspect of gaming remains important to her, nonetheless—and she still feels the need to connect with others. Achieving her personal goals and developing her skills in videogames brings her joy. Her time used for gaming has not increased during the pandemic. The interviewee's other hobbies included reading, mostly fantasy and sci-fi.

#18 Finnish Woman

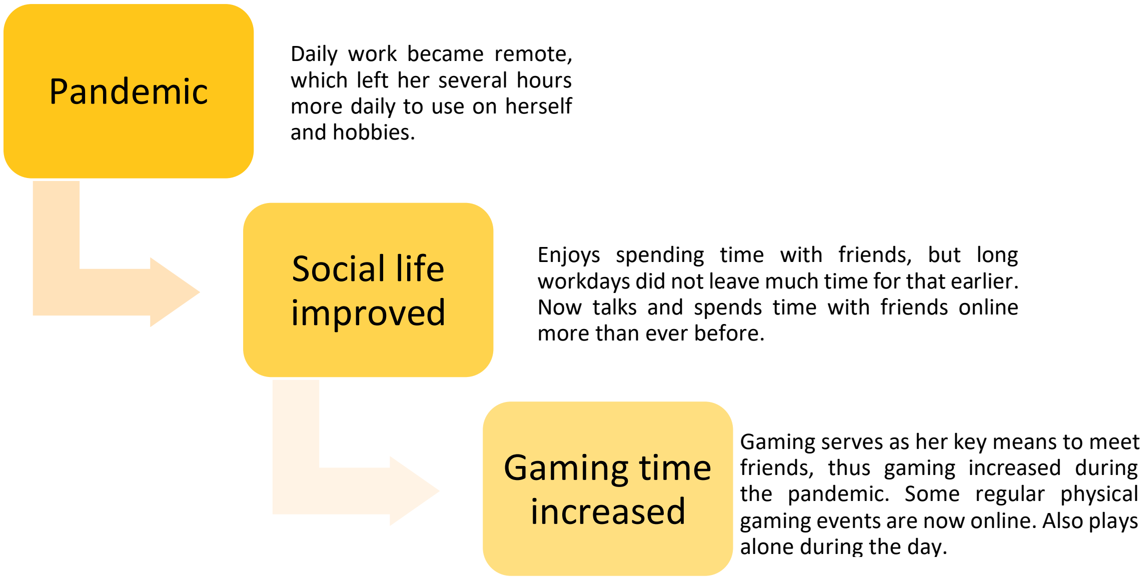

The interviewee describes her life during the pandemic as even better than before. She feels that her free time has increased, and she had more time to talk to her friends. She describes her feelings as calm and relaxed. The interviewee plays mobile games casually and infrequently, and multiplayer PC games more routinely. Her weekly gaming schedule had stayed mostly the same. Her most played game is Counter-Strike, which she plays with her friends. Gaming sessions are more about social interaction and hearing from others' lives than strictly about the videogame experience itself. She describes how she was emphatically and joyfully immersing herself in other players' triumphs. For that reason, she prefers to play with her friends instead of strangers. In her free time, the interviewee also watches TV and films, and has infrequent movie nights with her friends.

#19 Finnish Woman

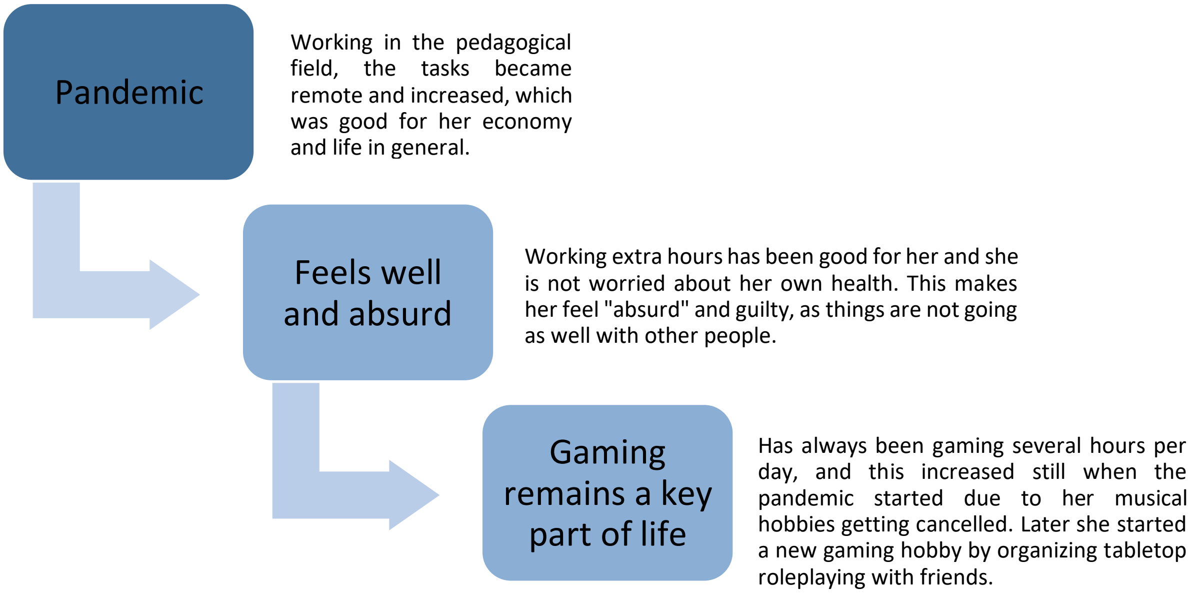

The interviewee describes her feelings about the pandemic “absurd.” She is concerned about her own health, but more about others'. Her physical social interactions have been few, but she keeps contact with her friends remotely. The interviewee plays several mobile and console games daily. She describes herself as being immersed in videogames and playing for several hours in a row. She also plays role-playing board games with her friends; she organizes it herself. Her playing habits have not changed much during the pandemic. In the summer, she started playing more tabletop games (D&D), about twice a month and acquiring new skills in it. Also, the time spent playing returned to pre-COVID-19 levels. She says that the most important aspects of playing with friends are the social interaction and mutual immersion, which give her feelings of contentment. To her, the most challenging thing as the game leader (DM) has been to create an environment where everyone enjoys themselves and gets their voice heard. Her musical hobbies are on a hold due to a cancellation of the choir sessions and living in an apartment building. Remote solutions have not worked out for practicing, only for maintaining social contacts. She also bakes actively in her free time.

#20 Finnish Man

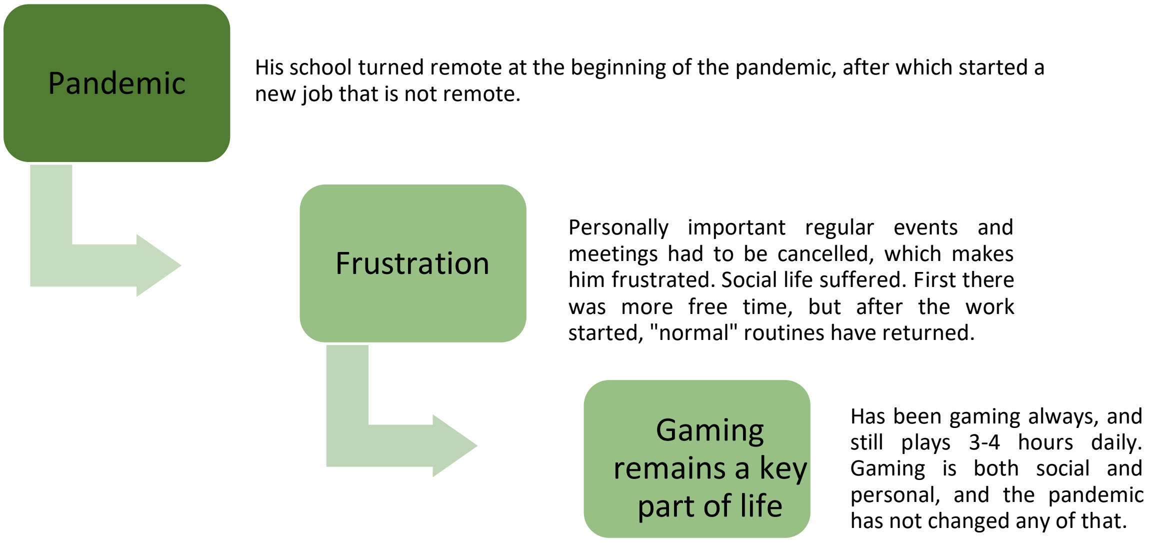

The interviewee expresses his frustration of the pandemic. His own life has not changed radically, only some meetings with his friends and relatives have been postponed and school is remote. The interviewee plays on PC and console games around 3-4 hours per weekday, usually more on the weekends. He plays multiplayer games only a little now compared to pre-COVID-19 times. He chooses his videogames spontaneously and switches between them during the same day. The videogames he plays currently are mostly the same as before the pandemic. He plays both alone and with friends. When he plays with friends, gaming is more about the social interaction. He would socialize with his friends on Discord even if they are playing different videogames. The interviewee also mentions that while playing with friends, he plays more seriously. In his free time, he likes to go jogging. He had not replaced the absence of social interactions with distant meetings.
